# The katanin A-subunits KATNA1 and KATNAL1 act co-operatively in mammalian meiosis and spermiogenesis to achieve male fertility

**DOI:** 10.1242/dev.201956

**Published:** 2023-11-13

**Authors:** Jessica E. M. Dunleavy, Maddison Graffeo, Kathryn Wozniak, Anne E. O'Connor, D. Jo Merriner, Joseph Nguyen, Ralf B. Schittenhelm, Brendan J. Houston, Moira K. O'Bryan

**Affiliations:** ^1^ School of BioSciences and Bio21 Institute, Faculty of Science, The University of Melbourne, Melbourne, VIC 3010, Australia; ^2^ Monash Biomedicine Discovery Institute and The Department of Anatomy and Developmental Biology, Monash University, Melbourne, VIC 3800, Australia; ^3^ Monash Proteomics & Metabolomics Facility, Department of Biochemistry and Molecular Biology, Biomedicine Discovery Institute, Monash University, Clayton, VIC 3800, Australia

**Keywords:** Katanin, Meiotic spindle, Spermatogenesis, Microtubule severing, Manchette, Axoneme, Acrosome, Head-to-tail coupling apparatus, Vesicle transport, Spermiation

## Abstract

Katanins, a class of microtubule-severing enzymes, are potent M-phase regulators in oocytes and somatic cells. How the complex and evolutionarily crucial, male mammalian meiotic spindle is sculpted remains unknown. Here, using multiple single and double gene knockout mice, we reveal that the canonical katanin A-subunit KATNA1 and its close paralogue KATNAL1 together execute multiple aspects of meiosis. We show KATNA1 and KATNAL1 collectively regulate the male meiotic spindle, cytokinesis and midbody abscission, in addition to diverse spermatid remodelling events, including Golgi organisation, and acrosome and manchette formation. We also define KATNAL1-specific roles in sperm flagellum development, manchette regulation and sperm-epithelial disengagement. Finally, using proteomic approaches, we define the KATNA1, KATNAL1 and KATNB1 mammalian testis interactome, which includes a network of cytoskeletal and vesicle trafficking proteins. Collectively, we reveal that the presence of multiple katanin A-subunit paralogs in mammalian spermatogenesis allows for ‘customised cutting’ via neofunctionalisation and protective buffering via gene redundancy.

## Teaser

**Summary:** The microtubule-severing enzymes KATNA1 and KATNAL1 function collectively to regulate multiple aspects of microtubule dynamics during spermatogenesis.

## INTRODUCTION

The katanins are an evolutionary conserved family of microtubule (MT)-severing proteins, first identified as essential meiosis regulators in *Caenorhabditis elegans* oocytes (Mains et al., 1990). Since then, they have been established as nuanced MT regulators, able to employ the same action (physical removal of tubulin heterodimers from the MT lattice) to amplify, disassemble or even strengthen MTs (McNally and Roll-Mecak, 2018; Sarbanes et al., 2022). At a cellular level, these actions drive rapid MT-remodelling events required in many processes, including ciliogenesis, cell division and differentiation, and allow fine tuning of homeostatic MT structures and processes.

The canonical katanin complex consists of the AAA ATPase catalytic enzyme KATNA1 (also known as p60 or the A-subunit) and the WD40 repeat-containing regulatory subunit KATNB1 (also known as p80 or the B-subunit). In higher eukaryotes, the B-subunit is not essential for A-subunit activity; however, it increases MT affinity and severing efficiency and is often essential for A-subunit localisation (McNally and Roll-Mecak, 2018). Paralogous A-subunits, KATNAL1 and KATNAL2, exist in vertebrates, *Drosophila* and unicellular ciliates, and an additional KATNB1 paralog lacking the WD40 domain, KATNBL1, exists in vertebrates. KATNAL1 is highly similar in sequence and domain architecture to KATNA1 (McNally and Roll-Mecak, 2018) and possesses MT-severing activity (Cheung et al., 2016). KATNAL2, however, outside of the conserved AAA ATPase MT-severing domain, differs in domain architecture (McNally and Roll-Mecak, 2018), and does not exhibit MT-severing activity in standard assays (Cheung et al., 2016; Dunleavy et al., 2017). This aside, KATNAL2 loss in mouse spermatogenesis results in phenotypes consistent with loss of MT severing (Dunleavy et al., 2017).

Across eukaryotes, regulation of cell division is a defining feature of katanin action. In *C. elegans* oocytes, the KATNA1 and KATNB1 orthologues MEI-1 and MEI-2 drive bipolar spindle formation via MT amplification and regulate spindle length, function and positioning in meiosis I (McNally and Roll-Mecak, 2018; Sharp and Ross, 2012), and mediate spindle disassembly in meiosis II (Gomes et al., 2013). KATNA1 also regulates female meiotic spindle length in *Xenopus laevis* and *Xenopus tropocalis* (Loughlin et al., 2011), and, in cultured mouse oocytes, KATNAL1 depletion results in abnormally long meiotic spindles and an increase in multipolar spindles (Gao et al., 2019). Likewise, in mitosis, katanin A-subunits have variously been shown to regulate mitotic spindle size/shape, polarity, density and orientation, and drive midbody abscission during cytokinesis in species ranging from unicellular ciliates and plants to mammalian cell lines (Lynn et al., 2021; McNally and Roll-Mecak, 2018; Sharp and Ross, 2012). Despite these data, the contribution of katanin proteins to male meiosis has received little attention. Loss-of-function studies of the regulatory B-subunit KATNB1 strongly suggest that katanin-mediated MT severing is required in male meiosis (Dunleavy et al., 2021; O'Donnell et al., 2012), and we have previously shown that the KATNB1-KATNA1, KATNB1-KATNAL1 and KATNB1-KATNAL2 complexes are present during male mammalian meiosis (Dunleavy et al., 2021). However, the requirement of katanin A-subunits has not been directly tested.

Herein, using a suite of single and double katanin A-subunit deletion mice, we reveal that KATNA1 and KATNAL1 collectively regulate male meiosis, wherein they refine spindle structure and function and regulate cytokinesis. We show that their functions extend into haploid germ cell development (spermiogenesis), wherein they restrain the global MT bulk and also have several structure/process-specific roles. We reveal that KATNAL1 fully functionally compensates for KATNA1 loss in male germ cells; however, KATNA1 only partially compensates for KATNAL1 loss, and no functional compensation exists between KATNA1 and KATNAL2. Finally, using an *in vivo* immunoprecipitation (IP)-mass spectrometry (MS) approach, we reveal that these diverse functions involve an equally diverse set of putative testis interacting partners, including MT- and actin-associated proteins, vesicle-transport proteins and essential mediators of male meiosis and acrosome biogenesis. These results reveal the complex reliance of spermatogenesis on katanin function and the precise neofunctionalisation of A-subunits required to achieve normal sperm function and morphology.

## RESULTS

### KATNA1 is not essential for male fertility in the mouse

To test the hypothesis that KATNA1 is the katanin A-subunit required in male meiosis, we generated a *Katna1* germ cell-specific knockout (GCKO) mouse model using *Stra8-Cre* (Sadate-Ngatchou et al., 2008) for pre-meiotic deletion of exons 6 and 7 of *Katna1* from male germ cells (*Katna1^GCKO/GCKO^*, Fig. S1A-C). Successful ablation of *Katna1* gene expression was confirmed by quantitative PCR (qPCR) and immunolabelling (Fig. S1D,E).

*Katna1^GCKO/GCKO^* mice exhibited normal body weight, testis weight, daily sperm production (DSP) and epididymal sperm content (Fig. S2A-D). Histological assessment of the *Katna1^GCKO/GCKO^* male reproductive tract revealed normal progression of germ cells through spermatogenesis and the presence of sperm in epididymides (Fig. S2E-G). *Katna1^GCKO/GCKO^* mouse sperm were cytologically comparable with controls (Fig. S2H) and were functionally competent, as demonstrated by normal fertility when mated with wild-type (WT) females [8.2±0.8 pups (mean±s.d.) per copulatory plug in *Katna1^GCKO/GCKO^* mice versus 7.1±0.7 pups in controls, *P*=0.0969, unpaired two-tailed *t*-test]. Collectively, these data reveal that KATNA1 is dispensable for mammalian spermatogenesis.

### Germ cell-derived KATNAL1 is required for optimal male fertility in mice

We next sought to explore the requirement during meiosis for the non-canonical katanin A-subunit KATNAL1. A role for KATNAL1 in the somatic Sertoli cells of the testis has been defined (Smith et al., 2012), but a role in male meiosis has not been tested. To do this, we generated a *Katnal1* GCKO mouse model again using *Stra8-Cre* (*Katnal1^GCKO/GCKO^*, Fig. S3A,B). Successful ablation of *Katnal1* gene expression was confirmed by qPCR, western blotting and immunolabelling (Fig. S3C-E).

**Fig. 1.**
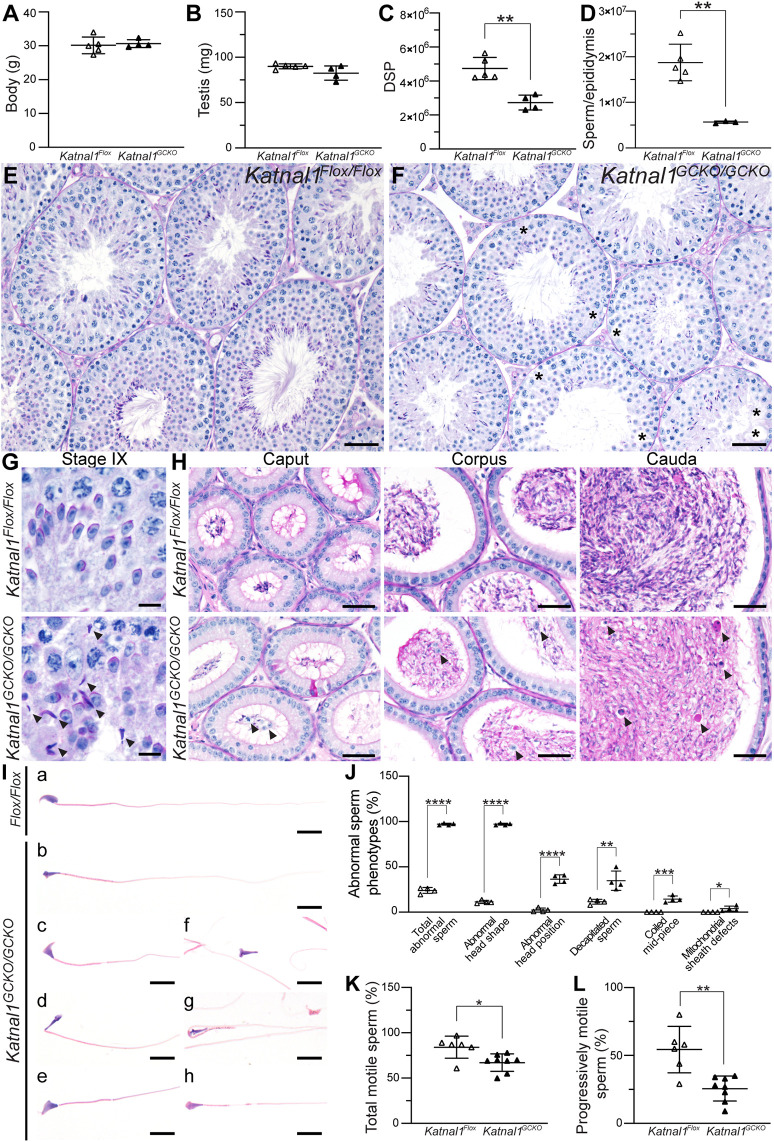
**Germ cell-derived KATNAL1 is required for optimal male fertility.** (A-D) Body weight (A), testis weight (B), daily sperm production (DSP) (C), and epididymal sperm content (D) in *Katnal1^Flox/Flox^* and *Katnal1^GCKO/GCKO^* mice (*n*≥3/genotype). (E-H) PAS-stained testis (E-G) and epididymis (H) sections. Asterisks (F) indicate areas with vacuoles or devoid of germ cells. Black arrowheads in G indicate retained spermatids. Black arrowheads in H indicate prematurely sloughed germ cells. (I) Haematoxylin and Eosin-stained cauda epididymal sperm. Abnormalities observed in *Katnal1^GCKO/GCKO^* mice included abnormal sperm head shapes – ‘knobby’ (b) and ‘hammer’ (c,e) head, abnormal sperm head position (d), decapitated sperm (f), coiled mid-pieces (g) and mitochondrial sheath defects (h). (J-L) Percentage of abnormal sperm phenotypes (J) and motile (K) and progressively motile (L) sperm from *Katnal1^Flox/Flox^* (white triangles) versus *Katnal1^GCKO/GCKO^* (black triangles) males (*n*≥3/genotype). For all histological analyses, a minimum of three mice/genotype were assessed and representative images are shown. Scale bars: 50 µm (E,F); 10 µm (G); 40 µm (H); 10 µm (I). For graphed data, lines represent mean±s.d. *P<0.05; **P<0.01; ***P<0.001; ****P<0.0001 (unpaired two-tailed *t*-test between genotypes for each phenotype).

*Katnal1^GCKO/GCKO^* males exhibited normal mating frequency but were subfertile, siring 84% fewer pups per copulatory plug than *Katnal1^Flox/Flox^* controls (6.9±3.1 pups/plug sired by *Katnal1^Flox/Flox^* males versus 1.4±1.6 s.d. pups/plug sired by *Katnal1^GCKO/GCKO^* males, *P*=0.0151, unpaired two-tailed *t*-test). *Katnal1^GCKO/GCKO^* mice had normal body and testis weights (Fig. 1A,B). Likewise, the number of apoptotic germ cells per seminiferous tubules was comparable with that of controls (Fig. S3F). Despite this, *Katnal1^GCKO/GCKO^* DSP was reduced by 42.3% compared with that of controls [(4.7±0.6)×10^6^ sperm (mean±s.d.) in *Katnal1^Flox/Flox^* versus (2.7±0.4)×10^6^ sperm in *Katnal1^GCKO/GCKO^*, *P*=0.0012, Fig. 1C], indicative of germ cell loss in the later steps of spermiogenesis when spermatids contribute minimally to overall testis weight. Finally, epididymal sperm content was reduced by 73.7% compared with that of controls [(1.8±0.4)×10^7^ sperm (mean±s.d.) in *Katnal1^Flox/Flox^* versus (5.7±0.2)×10^6^ sperm in *Katnal1^GCKO/GCKO^*, *P*=0.0016, Fig. 1D]. The more severe reduction in epididymal sperm content relative to DSP suggests a partial failure in sperm release from the seminiferous epithelium via spermiation.

Although all germ cell types were present in the *Katnal1^GCKO/GCKO^* seminiferous epithelium, many tubules contained fewer germ cells, notably spermatids, compared with those of controls (Fig. 1E,F). Gaps between adjacent Sertoli cells were frequently seen in *Katnal1^GCKO/GCKO^* mice but never in *Katnal1^Flox/Flox^* mice (Fig. 1E,F). The abnormal retention of elongated spermatids in *Katnal1^GCKO/GCKO^* stage IX tubules (Fig. 1G) confirmed frequent spermiation failure in *Katnal1^GCKO/GCKO^* mice. Epididymal histology was consistent with fewer sperm, in addition to the presence of prematurely sloughed germ cells, in *Katnal1^GCKO/GCKO^* mice but not in *Katnal1^Flox/Flox^* mice (Fig. 1H). Immunolabelling testis sections for the Sertoli cell-specific β-tubulin isoform TUBB3 confirmed that the origins of the reduced spermatogenic output in *Katnal1^GCKO/GCKO^* mice were distinct from those of the previously characterised *Katnal1^1H/1H^* hypomorphic mouse model, in which extensive disruption of Sertoli cell MT cytoskeleton was seen (Smith et al., 2012). In contrast, TUBB3 localisation in *Katnal1^GCKO/GCKO^* testis sections was comparable with that in *Katnal1^Flox/Flox^* testis sections (Fig. S4A,B). These data, taken together with that of Smith et al. (2012), reveal that KATNAL1 plays an essential role in MT regulation in both Sertoli and germ cells.

Of the sperm present in the *Katnal1^GCKO/GCKO^* mouse epididymides, almost all were morphologically abnormal [97.1±1.1% (mean±s.d.) in *Katnal1^GCKO/GCKO^* versus 23.9±3.2% in *Katnal1^Flox/Flox^*, *P*<0.0001, Fig. 1I,J]. The abnormalities included head shape (97.1±1.1% abnormal phenotypes in *Katnal1^GCKO/GCKO^* versus 11.3±1.7% in *Katnal1^Flox/Flox^*, *P*<0.0001), head position (36.4±4.7% versus 2.3±2.1%, *P*<0.0001), coupling between the head and tail (decapitation, 34.7±10.7% versus 11.5±2.7%, *P*=0.0056), mid-piece structure (coiling, 14.4±3.4% versus 0.0±0.0%, *P*=0.0002) and mitochondrial sheath structure (3.9±2.7% versus 0.0±0.0%, *P*=0.0294) (Fig. 1J). Moreover, of the sperm found in *Katnal1^GCKO/GCKO^* epididymides, there was a 20.2% reduction in total motility (*P*=0.0128) and a 52.9% reduction in progressive motility (*P*=0.0016) in the absence of KATNAL1 (Fig. 1K,L). Considering the reduction in sperm number in *Katnal1^GCKO/GCKO^* mice, this equates to an 87.6% reduction in the total number of progressively motile sperm compared with that in *Katnal1^Flox/Flox^* mice. Collectively, these phenotypes reveal that germ cell-derived KATNAL1 is required for normal sperm number, morphology and motility, and optimal male fertility.

### KATNAL1 regulates chromosome alignment, segregation and cytokinesis during male meiosis

**Fig. 2.**
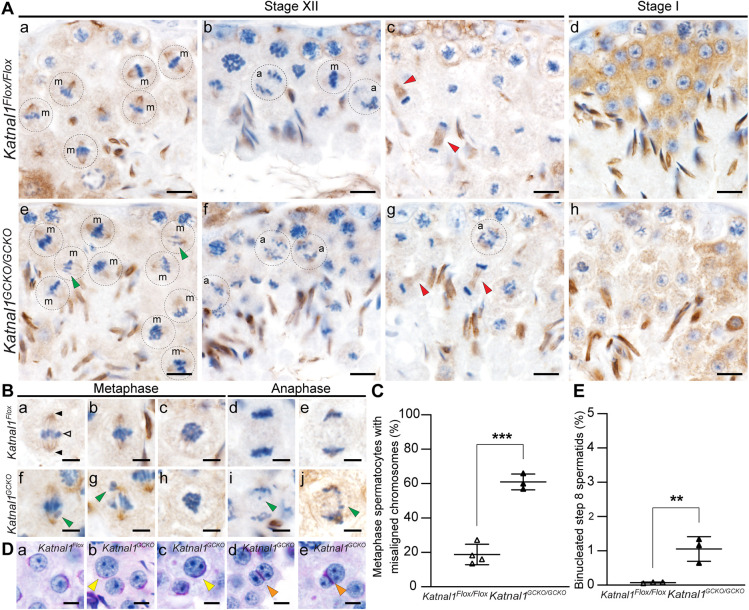
**KATNAL1 regulates chromosome alignment, segregation and cytokinesis during male meiosis.** (A,B) Testis sections immunolabelled for α-tubulin (MT marker). In A: m, metaphase spermatocytes; a, anaphase spermatocytes. Green arrowheads indicate misaligned/lagging chromosomes. Red arrowheads indicate midbodies. For reference in Ba, black arrowheads indicate spindle poles and the white arrowhead indicates chromosomes aligned at the metaphase plate. (C) Percentage of *Katnal1^Flox/Flox^* and *Katnal1^GCKO/GCKO^* metaphase spermatocytes with misaligned chromosomes in testis sections immunolabelled for α-tubulin (*n*≥3/genotype). (D) PAS-stained testis sections showing abnormally large round spermatid nuclei (yellow arrowheads in b,c) and binucleated round spermatids (orange arrowheads in d,e) in *Katnal1^GCKO/GCKO^* mice. (E) Percentage of step 8 spermatids in PAS-stained testis sections that were binucleated in *Katnal1^Flox/Flox^* and *Katnal1^GCKO/GCKO^* mice (*n*=3/genotype). Scale bars: 10 µm (A); 5 µm (B,D). For graphed data, lines represent mean±s.d. **P<0.01; ***P<0.001 (unpaired two-tailed *t*-test).

To assess *Katnal1^GCKO/GCKO^* meiotic spindles, testis sections were stained for α-tubulin (Fig. 2A,B, brown staining) and DNA was counterstained with Haematoxylin (Fig. 2A,B, blue staining). As seen in *Katnal1^Flox/Flox^* stage XII tubules, metaphase I and II meiotic spindle MTs typically project from the two opposing spindle poles in a narrow and uniform angle to tightly align chromosomes/chromatids along the metaphase plate (Fig. 2Aa,Ba-Bc). In *Katnal1^GCKO/GCKO^* stage XII tubules, the vast majority of metaphase spindles displayed normal spindle architecture and normal MT spindle density (Fig. 2Ae,Bf-Bh). Chromosomal alignment at the metaphase plate, however, was noticeably abnormal in 61±4.6% (±s.d.) of *Katnal1^GCKO/GCKO^* metaphase spermatocytes compared with 18.8±5.9% of *Katnal1^Flox/Flox^* controls (Fig. 2A,Bf,Bg,C, green arrowheads, *P*=0.0002). Despite these defects, no increase in metaphase arrest and apoptosis in stage XII to stage I seminiferous tubules was observed in *Katnal1^GCKO/GCKO^* mice compared with those in *Katnal1^Flox/Flox^* mice (Fig. 2A; Fig. S3F-L).

**Fig. 3.**
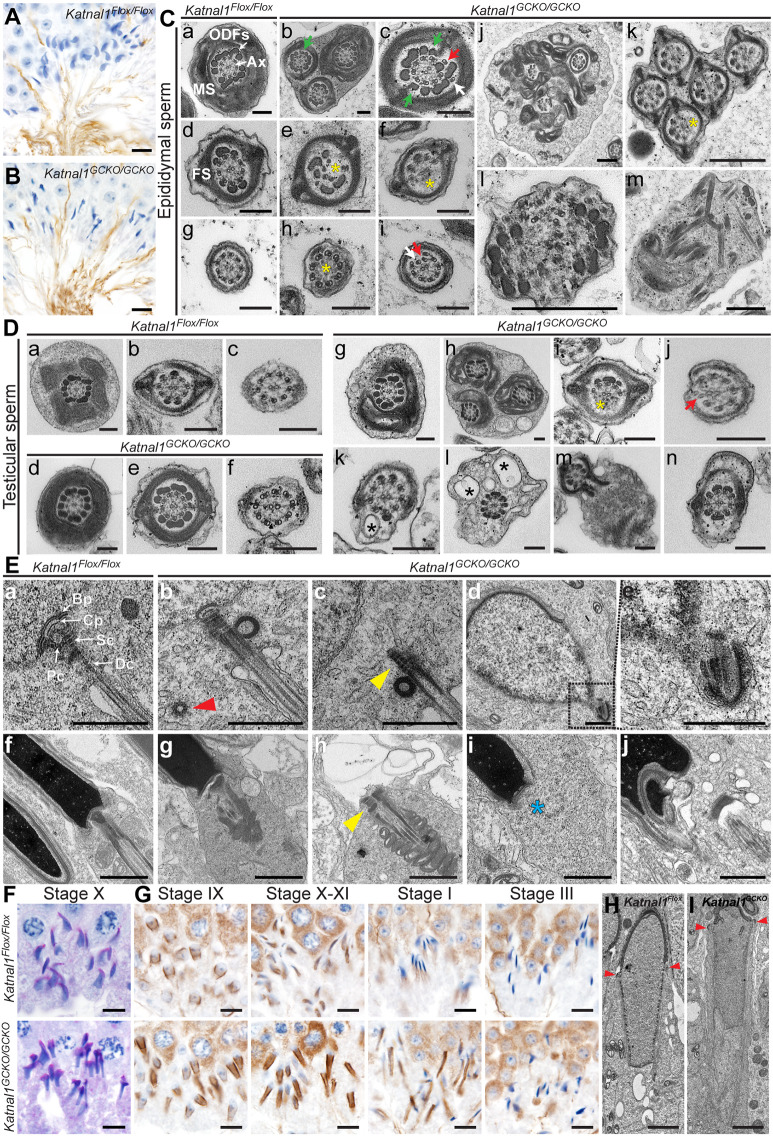
**KATNAL1 is a regulator of stable flagella and HTCA formation and the MT manchette in male germ cells.** (A,B) Testis sections immunolabelled for axonemes (acetylated tubulin) in *Katnal1^Flox/Flox^* (A) and *Katnal1^GCKO/GCKO^* (B) mice. (C,D) TEM images of cauda epididymal sperm flagella ultrastructure (C) and of developing spermatid flagella ultrastructure in (D) *Katnal1^Flox/Flox^* and *Katnal1^GCKO/GCKO^* mice. Ax, axoneme; FS, fibrous sheath; MS, mitochondrial sheath; ODFs, outer dense fibres. White arrows (Cc,Ci) indicate supernumerary MT doublets. Red arrows (Cc,Ci,Dj) indicate ectopic MT doublets. Yellow asterisks (Ce,Cf,Ch,Ck,Di) indicate missing axoneme MTs. Green arrows (Cb,Cc) indicate ectopic ODFs. Black asterisks (Dk,Dl) indicate ectopic vesicles. Mid-piece cross-sections are shown in Ca,Cb,Cj,Da,Dd,Dg,Dh, principal piece cross-sections are shown in Cb-f,Ck,De,Di,Dk,Dm,Dn, and end piece cross-sections are shown in Cg-i,Dc,Df,Dj. (E) TEM images of HTCA ultrastructure in round (a-e) and elongated (f-j) spermatids in *Katnal1^Flox/Flox^* (a,f) and *Katnal1^GCKO/GCKO^* (b-e,g-j) mice. Red arrowhead (b) indicates a supernumerary centriole. The blue asterisk (i) indicates an absent HTCA. Yellow arrowheads (c,h) indicate abnormal detachment of the HTCA from the nucleus. In a: Bp, basal plate; Cp, capitulum; Dc, distal centriole; Pc, proximal centriole; Sc, segmented columns. (F) PAS-stained testis sections showing *Katnal1^Flox/Flox^* and *Katnal1^GCKO/GCKO^* elongating spermatids. (G) *Katnal1^Flox/Flox^* and *Katnal1^GCKO/GCKO^* mouse testis sections immunolabelled for α-tubulin showing progressive steps of manchette formation, migration and dissolution from left to right. (H,I) TEM images showing manchette structure in *Katnal1^Flox/Flox^* and *Katnal1^GCKO/GCKO^* elongating spermatids. Red arrowheads indicate the perinuclear ring. For all histological analyses, a minimum of three mice/genotype were assessed and representative images are shown. Scale bars: 10 μm (A,B); 200 nm (Ca-i,D); 500 nm (Cj-m); 1 μm (E); 10 μm (F,G); 2 μm (H,I).

Similarly, there was no discernible increase in *Katnal1^GCKO/GCKO^* spermatocytes stalling in anaphase or telophase or undergoing apoptosis (Fig. 2A; Fig. S3F-L). Instead, anaphase and telophase defects in *Katnal1^GCKO/GCKO^* meiosis led to the production of abnormal haploid germ cells. In anaphase I and II, spindle MTs undergo poleward shortening, allowing them to pull chromosomes toward opposing poles in a coordinated manner, as observed in *Katnal1^Flox/Flox^* anaphase spermatocytes (Fig. 2Ab,Bd,Be). In *Katnal1^GCKO/GCKO^* spermatocytes, however, uneven segregation of chromosomes was often observed, with lagging chromosomes commonly seen (Fig. 2Af,Ag,Bi,Bj, green arrowheads). Consistent with uneven chromosome segregation, many round spermatids possessed abnormally large nuclei (Fig. 2Db,Dc, yellow arrowheads). During telophase and cytokinesis, midbodies were present in *Katnal1^GCKO/GCKO^* cells and were comparable with those in controls (Fig. 2Ac,Ag, red arrowheads). However, a subset of the resultant spermatids showed binucleation (Fig. 2Dd,De, orange arrowheads) and, as detailed below, sperm/spermatids with multiple axonemes (Fig. 3C) and supernumerary centrioles (Fig. 3Eb, red arrowhead) were often observed. Quantification of binucleated spermatids confirmed this, with 1.05±0.3% (±s.d.) of step 8 spermatids in *Katnal1^GCKO/GCKO^* mice being binucleated compared with 0.08±0.02% in *Katnal1^Flox/Flox^* mice (Fig. 2E, *P*=0.0092). This indicates that although midbodies formed in the absence of *Katnal1*, midbody remodelling into an intracytoplasmic bridge and the maintenance of individual daughter spermatids was disrupted.

### KATNAL1 regulates flagellum stability, head-to-tail coupling apparatus formation and manchette function in male germ cells

As detailed above, of the epididymal sperm produced in *Katnal1^GCKO/GCKO^* mice, 97.1% were morphologically abnormal, revealing that KATNAL1 is required during spermatid differentiation (spermiogenesis) (Fig. 1I,J). One of the first processes to commence in spermiogenesis is acrosome formation, and we recently identified katanin-mediated MT severing as a potential regulator of this process (Dunleavy et al., 2021). The identity of the involved MT-severing A-subunit remains unknown. Acrosome formation was overtly normal in *Katnal1^GCKO/GCKO^* mice compared with that in controls, indicating that KATNAL1 is not essential for this process (Fig. S4C).

Multiple abnormalities were, however, observed in *Katnal1^GCKO/GCKO^* mouse sperm tails and the head-to-tail coupling apparatus (HTCA), in addition to reduced sperm motility. Staining of *Katnal1^GCKO/GCKO^* testis sections for acetylated tubulin revealed that, similar to controls (Fig. 3A), axonemal MTs were present in sperm tails (Fig. 3B); however, analysis of mature epididymal sperm via transmission electron microscopy (TEM) revealed a range of axoneme and accessory structure abnormalities (Fig. 3C). In *Katnal1^GCKO/GCKO^* mice, epididymal sperm flagellum axonemes often lacked the typical 9+2 MT doublet arrangement observed in controls (Fig. 3Ca,Cd,Cg). Instead, MT doublets were often missing (Fig. 3Ce,Cf,Ch, yellow asterisks) or ectopically located (Fig. 3C, red arrows), or supernumerary MT doublets were present (Fig. 3Cc,Cl, white arrows). Consistent with cytokinesis failures, multiple axonemes, often each with associated accessory structures, were frequently observed within one cell (Fig. 3Cb,Cj-Cl). Abnormalities in MT doublet location and number were typically accompanied by defects in outer dense fibre (ODF) location and number (Fig. 3C). In control sperm, the mid-piece contains nine ODFs closely associated with the nine outer MT doublets of the axoneme (Fig. 3Ca). In the principal piece, ODFs 3 and 8 are replaced by the longitudinal columns of the fibrous sheath (Fig. 3Cd). In *Katnal1^GCKO/GCKO^* mice, however, sperm tail ODFs 3 and/or 8 frequently persisted into the principal piece (Fig. 3Cb,Cc, green arrowheads). In *Katnal1^GCKO/GCKO^* mouse sperm tails, in which some or all axoneme MTs were missing, ODFs were often also missing (Fig. 3Ce,Cf). Consistent with the coiled mid-pieces and mitochondrial sheath defects in some sperm from *Katnal1^GCKO/GCKO^* mice, TEM revealed that the mitochondria were not properly organised into a helical sheath in many sperm (Fig. 3Cj). Finally, in some sperm, only disorganised fragments of sperm tail components were observed, indicative of degenerating cells (Fig. 3Cm).

To determine whether these defects were evident during flagellar development, we examined testis sections via TEM (Fig. 3D). This revealed that the majority of spermatids in *Katnal1^GCKO/GCKO^* testes developed axonemes with the typical 9+2 MT doublet arrangement and visually normal accessory structures (ODFs, mitochondrial sheath and fibrous sheath) (Fig. 3Dd-Df). In a small subset of flagella, abnormalities similar to those observed in the subsequent epididymal sperm were seen, including abnormal mitochondrial loading (Fig. 3Dg,Dh), multiple axonemes within one cell (Fig. 3Dh), missing axonemal MT doublets and ODFs (Fig. 3Di, yellow asterisk), and ectopic axonemal doublets (Fig. 3Dj, red arrow). In addition, ectopic vesicles were observed in many flagella (Fig. 3Dk,Dl, black asterisks). These abnormalities were less common than in the epididymal sperm of *Katnal1^GCKO/GCKO^* mice, suggesting that although KATNAL1 is not required for the development of a visually normal sperm flagellar structure, it is required for the formation of a stable flagellum. In the absence of KATNAL1, and in the presence of the shearing forces sperm experience during transit through the excurrent ductal system (Chan and Hirashima, 2022), sperm lose structural integrity. Our data also suggest that KATNAL1 contributes to the development of the mitochondrial sheath.

The frequent decapitation or hyper-extension of sperm heads in sperm from *Katnal1^GCKO/GCKO^* males indicates that the HTCA was weak and/or improperly formed. TEM of round and elongated spermatids revealed a range of HTCA abnormalities in *Katnal1^GCKO/GCKO^* mice (Fig. 3E). In *Katnal1^Flox/Flox^* mice, spermatid HTCAs were easy to find in TEM testis sections. As expected, they contained a proximal centriole coupled to the nucleus via the basal plate and capitulum, a distal centriole coupled to the plasma membrane and continuous with the axoneme, and the surrounding segmented columns that are continuous with the ODFs (Fig. 3Ea,Ef). In *Katnal1^GCKO/GCKO^* mice, however, normal HTCAs were rare (Fig. 3Eb,Eg) and most were abnormal (Fig. 3Ec-Ee,Eh-Ej). In some cells, the HTCA and associated axoneme were present but not attached to the nucleus (Fig. 3Ec,Eh, yellow arrowheads), whereas in others, the basal plate could be seen associated with the nucleus, but the rest of the HTCA was absent (Fig. 3Eg). Quantification revealed that, of the HTCAs that were visible via TEM, 64±31.1% (±s.d.) were abnormal in *Katnal1^GCKO/GCKO^* mice compared with 0±0% in *Katnal1^Flox/Flox^* (*P*=0.0232, unpaired two-tailed *t*-test, *n*=3/genotype). Consistent with these data, immunolabelling of sperm for SUN5, an essential component of the linker of nucleoskeleton and cytoskeleton (LINC) complex that physically docks the sperm centrosome to the nucleus, revealed a 57.2% reduction in sperm containing SUN5 at the HTCA in *Katnal1^GCKO/GCKO^* males compared with those in controls (*P*=0.0021, Fig. S4D,E). These data suggest that KATNAL1-regulated MTs are required either to fortify the basal plate and the nuclear connection or for the delivery and deposition of essential HTCA components.

Abnormal head shape was the most frequent morphological abnormality present in *Katnal1^GCKO/GCKO^* mouse sperm. Instead of the normal hook shape exhibited in 88.75% of control sperm, 34% of *Katnal1^GCKO/GCKO^* mouse sperm heads exhibited a ‘hammerhead’ shape and 62% exhibited a ‘knobby’ head phenotype (Fig. 1I; Fig. S4F,G). Abnormal head shaping became apparent during nuclear elongation (Fig. 3F) and was consistent with defective function of the manchette, a transient MT-based structure that sculpts the distal half of the spermatid head. As shown in Fig. 3G, in control mice, the manchette forms in step 8 to step 9 spermatids (stage VIII to stage IX seminiferous tubules), before migrating distally and simultaneously constricting to shape the spermatids head during steps 10-13 (stage X to stage I; Fig. 3G). In steps 13 and 14 (stages II and III), the manchette is disassembled (Fig. 3G)*.* In *Katnal1^GCKO/GCKO^* mouse spermatids, manchettes formed at steps 8 to 9 but were abnormally elongated. Elongation became increasingly pronounced as spermiogenesis continued and was associated with a failure of manchette perinuclear ring migration down the sperm head, but the manchette continued constriction, resulting in a ‘knobby head’ phenotype (Fig. 3G). TEM analysis confirmed the absence of manchette migration (Fig. 3H,I). Finally, manchette disassembly in steps 13 and 14 was delayed in the absence of KATNAL1 (Fig. 3G). Collectively, these results reveal that KATNAL1-mediated MT severing is required to regulate manchette MT length, migration and disassembly.

### KATNA1 and KATNAL1 compensate for each other in male meiosis and spermiogenesis

Given the apparently normal fertility in *Katna1^GCKO/GCKO^* mice and residual fertility in *Katnal1^GCKO/GCKO^* mice, we sought to explore the possibility of functional compensation between KATNA1 and its paralogs KATNAL1 and KATNAL2 in the testis. This is a particularly crucial question for meiosis wherein the identity of the crucial A-subunit(s) remains unknown. As reported previously, *Katnal2* loss has no apparent effect on male meiosis but does result in male infertility due to severe sperm developmental defects (Dunleavy et al., 2017). To test for redundancy, we generated *Katna1* and *Katnal1* double germ cell knockout mice (*Katna1/al1^GCKO/GCKO^*), and double *Katna1* germ cell and *Katnal2* knockout mice (*Katna1^GCKO/GCKO^/al2^KO/KO^*). The *Katna1^GCKO/GCKO^/al2^KO/KO^* mouse model (Fig. S5) phenocopied our previously characterised *Katnal2* mutant models (Dunleavy et al., 2017), including having a comparable spermiation failure phenotype, sperm head-shaping defects (knobby heads), and an absence of normal sperm tail development (Fig. S5). No aspect of the infertility phenotype appeared worse than loss of KATNAL2 in isolation (Dunleavy et al., 2017), revealing that KATNA1 and KATNAL2 do not have compensatory function in male germ cell development.

**Fig. 4.**
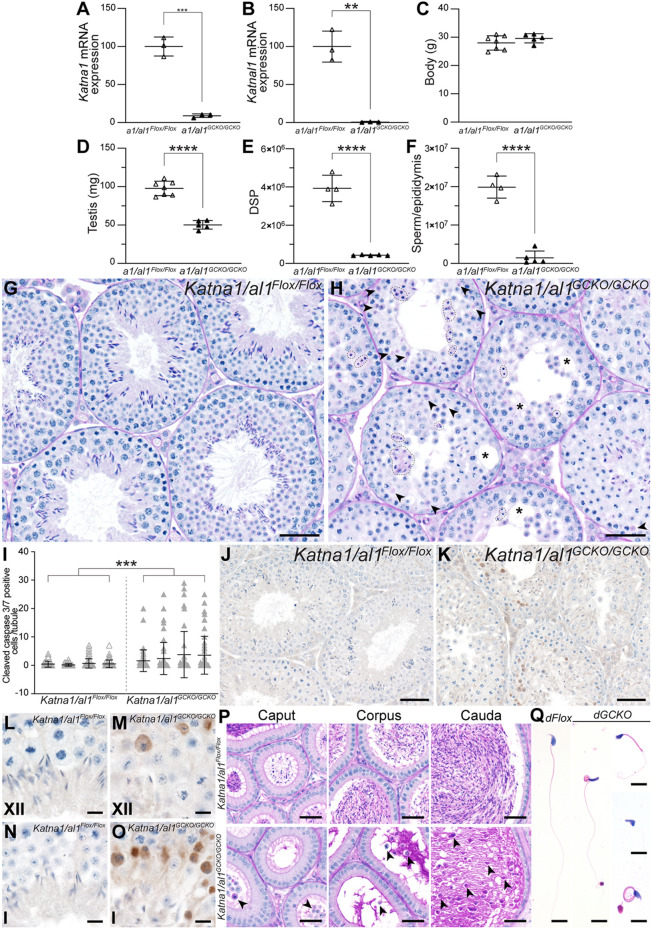
**Double germ cell deletion of KATNA1 and KATNAL1 results in a severe oligozoospermia-like phenotype.** (A,B) qPCR analysis of *Katna1* (A) and *Katnal1* (B) transcript levels in *Katna1*/*al1^Flox/Flox^* and *Katna1/al1^GCKO/GCKO^* isolated spermatocytes (*n*≥3/genotype). (C-F) Body weight (C), testis weight (D), testis DSP (E) and epididymal sperm content (F) in *Katna1/al1^Flox/Flox^* and *Katna1/al1^GCKO/GCKO^* mice (*n*≥3/genotype). Lines represent mean±s.d. (G,H) PAS-stained testis sections from *Katna1/al1^Flox/Flox^* and *Katna1/al1^GCKO/GCKO^* mice. Black arrowheads indicate pyknotic spermatocytes. Pyknotic spermatids are outlined by dashed lines. Asterisks indicate areas with vacuoles or areas devoid of germ cells. (I-O) Analysis of germ cell apoptosis in *Katna1/al1^Flox/Flox^* and *Katna1/al1^GCKO/GCKO^* mice. The average number of cleaved caspase 3/7-positive germ cells per seminiferous tubule per mouse is graphed in I and representative images are shown in J-O. In I, lines represent mean±s.d. A minimum of 50 tubules per mouse were counted (*n*≥3/genotype). In L-O, Roman numerals denote the seminiferous tubule stage. (P,Q) PAS-stained epididymis sections (P) and Haematoxylin and Eosin-stained cauda epididymal sperm (Q) from *Katna1/al1^Flox/Flox^* (*dFlox*) and *Katna1/al1^GCKO/GCKO^* (*dGCKO*) mice. Arrowheads in P indicate prematurely sloughed germ cells. For all histological analyses, a minimum of three mice/genotype were assessed and representative images are shown. Scale bars: 50 μm (G,H,J,K); 10 μm (L-O,Q); 40 μm (P). *P<0.05; **P<0.01; ***P<0.001; ****P<0.0001. Statistical significance was determined in A-F using an unpaired two-tailed *t*-test and in I with a generalised linear mixed model as detailed in the Materials and Methods.

By contrast, double germ cell knockout of *Katna1* and *Katnal1* resulted in a significantly worse male fertility phenotype than seen in the single *Katnal1* germ cell knockout mice, especially in meiosis. Ablation of both *Katna1* and *Katnal1* from male germ cells was confirmed by qPCR (Fig. 4A,B) and mice were overtly healthy, with normal body weight compared with controls (Fig. 4C). Test mating of *Katna1/al1^GCKO/GCKO^* male mice with WT females revealed that loss of both KATNA1 and KATNAL1 from male germ cells resulted in male sterility [7.1±3.2 pups/plug (mean±s.d.) sired by *Katna1/al1^Flox/Flox^* males versus 0±0 pups/plug sired by *Katna1/al1^GCKO/GCKO^* males, *P*=0.0175, unpaired two-tailed *t*-test]. Assessment of the reproductive tract revealed a 48.5% reduction in testis weight [97.7±9.3 mg (±s.d) in *Katna1/al1^Flox/Flox^* versus 50.3±5.63 mg in *Katna1/al1^GCKO/GCKO^*, *P*<0.0001], an 89% reduction in DSP [(3.9±0.63)×10^6^ sperm (±s.d.) versus (4.0±0.2)×10^5^ sperm, *P*<0.0001] and a 96.2% reduction in epididymal sperm content [(2.0±0.3)×10^7^ sperm (±s.d.) versus (1.5±1.8)×10^6^ sperm, *P*<0.0001] (Fig. 4D-F). Examination of *Katna1/al1^GCKO/GCKO^* testis histology instantly revealed large-scale germ cell loss during both meiosis and spermiogenesis that was significantly worse than in either single gene deletion (Fig. 4G,H). Numerous pyknotic spermatocytes were observed in all stage XII and stage I *Katna1/al1^GCKO/GCKO^* tubules, indicative of cells stalling in meiosis, followed by apoptosis, but these were rare in *Katna1/al1^Flox/Flox^* tubules (Fig. 4G,H, black arrowheads). Of the germ cells that progressed through meiosis in *Katna1/al1^GCKO/GCKO^*, the vast majority became pyknotic by steps 12 or 13 (stages XII and I) of elongating spermatid development (Fig. 4H, outlined by dashed lines) and, as a result, elongated spermatids were absent beyond stages II-VIII (Fig. 4H). Analysis of cleaved caspase 3/7 immunolabelling revealed a 6.4-fold increase in the number of apoptotic germ cells in *Katna1/al1^GCKO/GCKO^* versus *Katna1/al1^Flox/Flox^* seminiferous tubules (*P*=0.00103, Fig. 4I). The majority of apoptotic cells were spermatocytes that had stalled in anaphase I and meiosis II in stage I tubules (Fig. 4J-O) and, to a lesser degree, those undergoing metaphase I in stage XII (Fig. 4M). Analysis of epididymal sections confirmed almost no sperm reached the *Katna1/al1^GCKO/GCKO^* epididymis. The bulk of the cells in the lumen of *Katna1/al1^GCKO/GCKO^* epididymides were instead prematurely sloughed round germ cells (Fig. 4P, arrowheads). Of the few sperm present in the *Katna1/al1^GCKO/GCKO^* epididymides, all were morphologically abnormal, with abnormalities in sperm head shape, head position, head-to-tail attachment (decapitation) and the sperm tail (mid-piece coiling and mitochondrial sheath defects) (Fig. 4Q). The minimal numbers of epididymal sperm in this model precluded motility assessment.

**Fig. 5.**
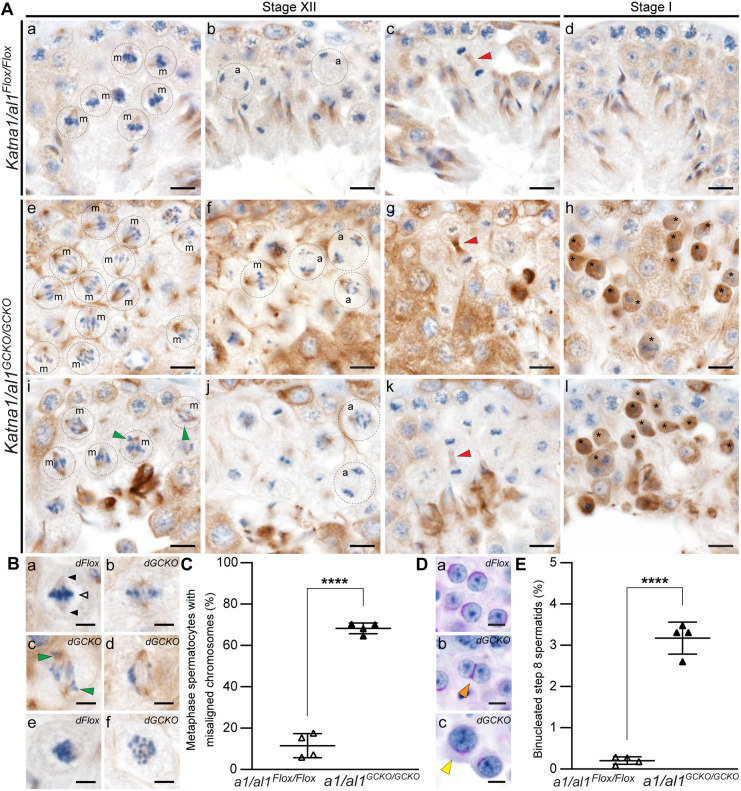
**KATNA1 and KATNAL1 compensate for each other in male meiosis.** (A,B) *Katna1/al1^Flox/Flox^* (*dFlox*) and *Katna1/al1^GCKO/GCKO^* (*dGCKO*) testis sections immunolabelled for MTs (α-tubulin). Nuclei were counterstained with Haematoxylin. Green arrowheads (Ai,Bc) indicate misaligned/lagging chromosomes. Red arrowheads (Ac,Ag,Ak) indicate midbodies. m, metaphase spermatocytes; a, anaphase spermatocytes. For reference in Ba, black arrowheads indicate spindle poles and the white arrowhead indicates chromosomes aligned at the metaphase plate. (C) Percentage of *Katna1/al1^Flox/Flox^* and *Katna1/al1^GCKO/GCKO^* metaphase spermatocytes with misaligned chromosomes in testis sections immunolabelled for α-tubulin (*n*=4/genotype). (D) PAS-stained *Katna1/al1^Flox/Flox^* (*dFlox*) and *Katna1/al1^GCKO/GCKO^* (*dGCKO*) testis sections. The orange arrowhead indicates a binucleated spermatid. The yellow arrowhead indicates an abnormally large round spermatid nucleus. (E) Percentage of step 8 spermatids in PAS-stained testis sections that were binucleated in *Katna1/al1^Flox/Flox^* and *Katna1/al1^GCKO/GCKO^* mice (*n*=4/genotype). Scale bars: 10 μm (A); 5 μm (B,D). For graphed data, lines represent mean±s.d. ****P<0.0001 (unpaired two-tailed *t*-test).

Assessment of *Katna1/al1^GCKO/GCKO^* meiotic spindles revealed that although meiotic spindles formed in *Katna1/al1^GCKO/GCKO^* spermatocytes, most were morphologically abnormal compared with those in *Katna1/al1^Flox/Flox^* controls (Fig. 5A,B). At metaphase, in contrast to the tightly packed morphology of the chromosomes in *Katna1/al1^Flox/Flox^* controls (Fig. 5A,Ba,Be), chromosomes were loosely dispersed along the metaphase plate in *Katna1/al1^GCKO/GCKO^* mice (Fig. 5A,Bb,Bf). Misaligned and/or lagging chromosomes were frequently observed in *Katna1/al1^GCKO/GCKO^* metaphase and anaphase spermatocytes [Fig. 5A-C, green arrowheads, 68.2±2.6% (±s.d.) of *Katna1/al1^GCKO/GCKO^* metaphase chromosomes were misaligned compared with 11.5±5.8% of *Katna1/al1^Flox/Flox^* metaphase chromosomes, *P*<0.0001]. As detailed above, these defects ultimately led to a large proportion of *Katna1/al1^GCKO/GCKO^* metaphase and anaphase spermatocytes stalling and undergoing apoptosis in stage XII to stage I seminiferous tubules (Fig. 4H-O). Examination of *Katna1/al1^GCKO/GCKO^* stage I tubules revealed that these stalled metaphase and anaphase spermatocytes were characterised by ectopic α-tubulin accumulation throughout their cytoplasm (Fig. 5Ah,Ai, asterisks) indicating that KATNA1/KATNAL1-mediated severing restrains the spermatocyte MT bulk. Of the *Katna1/al1^GCKO/GCKO^* spermatocytes that progressed through meiosis, overtly normal midbody formation was observed (Fig. 5Ag,Ak, red arrowheads). However, indicative of failure of midbody to intracellular bridge conversion, binucleated spermatids were frequently observed in *Katna1/al1^GCKO/GCKO^* mice but were rare in *Katna1/al1^Flox/Flox^* controls (Fig. 5Da,Db). Quantification revealed that 3.2±0.4% (±s.d.) of step 8 spermatids in *Katna1/al1^GCKO/GCKO^* mice were binucleated compared with 0.2±0.1% in *Katna1/al1^Flox/Flox^* mice (Fig. 5E, *P*<0.0001). This increase in the frequency of binucleated spermatids in *Katna1/al1^GCKO/GCKO^* mice was 3-fold more severe than that seen in *Katnal1^GCKO/GCKO^* mice. Consistent with compromised chromosome segregation, abnormally large round spermatids were frequent in *Katna1/al1^GCKO/GCKO^* mice but never seen in controls (Fig. 5D, yellow arrowhead). Collectively, these data indicate that KATNAL1 and KATNA1 can at least partially compensate for each other in male meiosis.

**Fig. 6.**
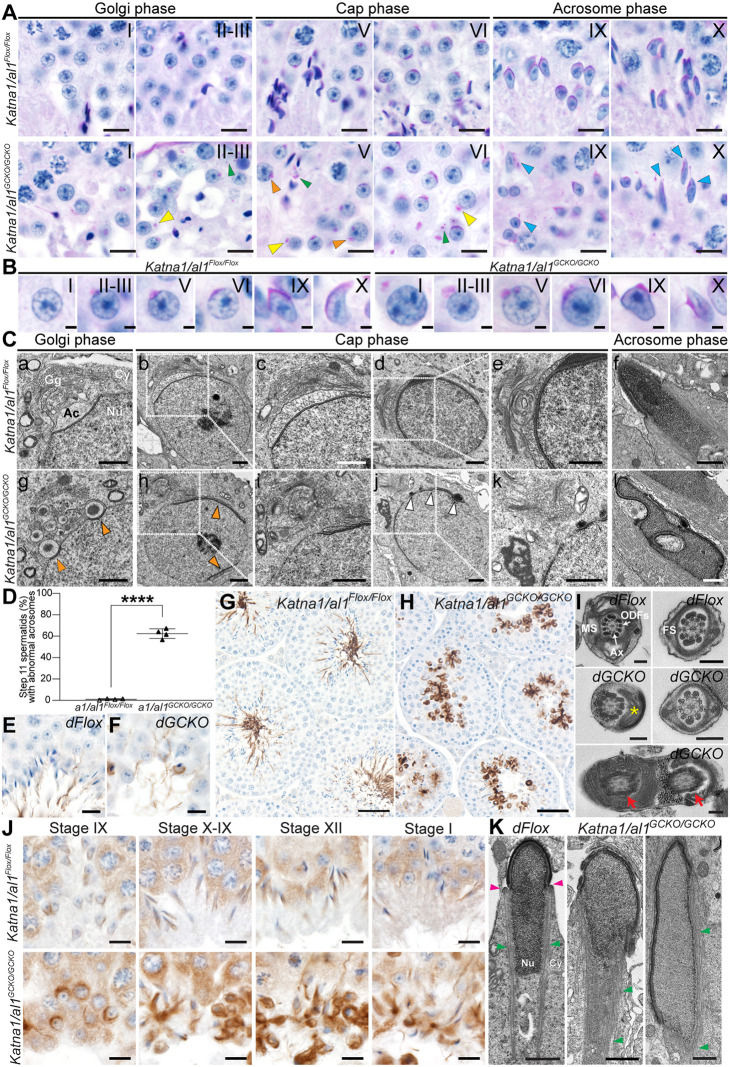
**KATNA1 and KATNAL1 compensation in spermatid acrosome formation and MT regulation.** (A,B) PAS-stained testis sections showing progressive steps of acrosome formation from left to right in *Katna1/al1^Flox/Flox^* and *Katna1/al1^GCKO/GCKO^* mice. Roman numerals denote seminiferous tubule stages. Orange arrowheads indicate nuclei with more than one proacrosomal vesicle attached. Yellow arrowheads indicate acrosomal vesicles not attached to the nucleus. Green arrowheads indicate ectopic PAS-positive vesicles abnormally dispersed in cytoplasm. Blue arrowheads indicate elongating spermatids with abnormal gaps/fragmentation of the acrosomes. (C) Electron micrographs showing acrosome and Golgi apparatus ultrastructure in *Katna1/al1^Flox/Flox^* and *Katna1/al1^GCKO/GCKO^* mice. Orange arrowheads indicate supernumerary sites of acrosome formation. White arrowheads indicate numerous small unfused acrosomal vesicles docked at the nuclear membrane/fragmentation of the acrosome. (D) Percentage of step 11 spermatids in PAS-stained testis sections with acrosome abnormalities in *Katna1/al1^Flox/Flox^* and *Katna1/al1^GCKO/GCKO^* mice (*n*=4/genotype). Lines represent mean±s.d. ****P<0.0001 (unpaired two-tailed *t*-test). (E-H) *Katna1/al1^Flox/Flox^* (*dFlox*) and *Katna1/al1^GCKO/GCKO^* (*dGCKO*) mouse testis sections immunolabelled for acetylated tubulin as a marker of the axoneme and stable MTs. (I) TEM images of developing flagellum ultrastructure in spermatids of *Katna1/al1^Flox/Flox^* (*dFlox*) and *Katnal1^GCKO/GCKO^* (*dGCKO*) mice. Ax, axoneme; FS, fibrous sheath; MS, mitochondrial sheath; ODFs, outer dense fibers. The yellow asterisk indicates an abnormal mitochondrial sheath. Red arrows indicate two flagella within one cell. (J) *Katna1/al1^Flox/Flox^* and *Katna1/al1^GCKO/GCKO^* testis sections immunolabelled for α-tubulin showing progressive steps of manchette formation, migration and dissolution from left to right. (K) TEM images showing nuclear and manchette morphology in *Katna1/al1^Flox/Flox^* (*dFlox*) and *Katnal1^GCKO/GCKO^* mouse elongating spermatids. Pink arrowheads indicate the perinuclear ring. Green arrowheads indicate MTs. For all histological analyses, a minimum of three mice/genotype were assessed and representative images are shown. Scale bars: 10 μm (A,E,F,J); 200 nm (B,I); 1 μm (C,K); 50 μm (G,H). Ac, acrosome; Cy, cytoplasm; Gg, Golgi apparatus; Nu, nucleus.

Assessment of *Katna1/al1^GCKO/GCKO^* mouse spermiogenesis revealed that unlike the single GCKO mice, acrosome biogenesis was overtly abnormal, indicating functional compensation between KATNA1 and KATNAL1 (Fig. 6A,B). In periodic acid-Schiff (PAS)-stained control testis sections, a single PAS-positive acrosomal vesicle adhered to step 2-3 round spermatid nuclei (stage II-III) in the Golgi phase of acrosome development, and progressively spread to cover the apical half of the spermatid nuclei as spermiogenesis progressed (Fig. 6A,B). In *Katna1/al1^GCKO/GCKO^* mice, however, a large subset of round spermatids with supernumerary ectopic acrosomal vesicles was observed (Fig. 6A,B, orange arrowheads), in addition to a smaller subset wherein pro-acrosomal vesicles had failed to attach to the nucleus at the appropriate time (Fig. 6A,B, yellow arrowheads). As spermiogenesis progressed, elongating spermatids with fragmented and partially detached acrosomes were common in *Katna1/al1^GCKO/GCKO^* mice, but never seen in controls (Fig. 6A,B, blue arrowheads). Ectopic PAS-positive vesicles were also present throughout the seminiferous epithelium during multiple steps of acrosome biogenesis in *Katna1/al1^GCKO/GCKO^* mice but not in *Katna1/al1^Flox/Flox^* mice (Fig. 6A, green arrowheads). TEM analysis confirmed these observations (Fig. 6C) and revealed morphological abnormalities in the spermatid Golgi apparatus of *Katna1/al1^GCKO/GCKO^* mice (Fig. 6Ci,Ck) compared with that in *Katna1/al1^Flox/Flox^* mice (Fig. 6Cc,Ce). In control round spermatids (Fig. 6Cc,Ce), the Golgi apparatus typically has a flattened, sometimes horseshoe-like, morphology wherein the trans-Golgi network is closely associated with and oriented in parallel to the apical nuclear membrane. In *Katna1/al1^GCKO/GCKO^* mice, however, the Golgi stacks were disorganised, often displaying a circular morphology wherein the cis-Golgi network almost entirely enclosed the trans-Golgi network (Fig. 6Ci), or a dispersed, splayed morphology (Fig. 6Ck). Finally, quantification of acrosome defects in PAS-stained seminiferous tubule cross-sections revealed that 62.4±4.5% (±s.d.) of step 11 *Katna1/al1^GCKO/GCKO^* mouse spermatids had detectable acrosome abnormalities, compared with 1.0±0.6% in *Katna1/al1^Flox/Flox^* (Fig. 6D, *P*<0.0001). These phenotypes reveal that KATNA1 and KATNAL1 collectively regulate the spatial organisation of the Golgi apparatus, and proacrosomal vesicle transport and fusion to the nuclear membrane. The *Katna1/al1^GCKO/GCKO^* mouse acrosome abnormalities are reminiscent of those seen in KATNB1 loss-of-function mice (Dunleavy et al., 2021), suggesting that KATNA1 and KATNAL1 likely function in complexes with KATNB1 during acrosome biogenesis.

Analysis of the key MT-based structures required for spermiogenesis revealed phenotypes consistent with a loss of MT severing and global dysregulation of the MT cytoskeleton. Similar to *Katna1^GCKO/GCKO^* mice, sperm tails formed in *Katna1/al1^GCKO/GCKO^* mice; however, reflective of sperm numbers, fewer tails were seen than in controls (Fig. 6E-H). Acetylated tubulin staining, however, revealed the frequent ectopic accumulation of stable MTs in the cytoplasm of *Katna1/al1^GCKO/GCKO^* mouse spermatids (Fig. 6H), consistent with decreased MT turnover. TEM assessment of developing spermatid flagella confirmed that axonemes developed in *Katna1/al1^GCKO/GCKO^* mice (Fig. 6I). Similar to *Katnal1^GCKO/GCKO^*, flagella with abnormal mitochondrial sheaths (Fig. 6I, yellow asterisk) and the presence of multiple flagella within one cell were observed (Fig. 6I, red arrows). The minimal epididymal sperm numbers in this model precluded TEM analysis of mature sperm flagella.

Immunolabelling of testis sections for α-tubulin revealed a catastrophic dysregulation of manchette formation. In *Katna1/al1^GCKO/GCKO^* mice, although α-tubulin began to accumulate within the cytoplasm of round spermatids from stage IX, coincident with when the manchette normally develops (Fig. 6J), it failed to assemble into a MT sheath around the distal half of the spermatid nucleus. Instead, the cytoplasm of the *Katna1/al1^GCKO/GCKO^* mouse spermatids became enriched in ectopic α-tubulin as spermiogenesis progressed (Fig. 6J). TEM analysis of elongating spermatid ultrastructure confirmed these observations (Fig. 6K). In control spermatids, a typical manchette structure wherein MTs projected distally from a perinuclear ring was observed (Fig. 6K). In *Katna1/al1^GCKO/GCKO^* mice, MTs were observed throughout the cytoplasm of elongating spermatids; however, these were never associated with a perinuclear ring and were not arranged into a typical manchette structure (Fig. 6K). Collectively, these results reveal that KATNA1/KATNAL1-mediated severing is required to restrain MT growth/prune the MT bulk during spermatid differentiation and to regulate manchette formation. Moreover, the absence of these phenotypes in the single GCKO models suggest that KATNA1 and KATNAL1 can compensate for each other in these aspects of spermiogenesis.

Finally, to explore the mechanisms of KATNA1 and KATNAL1 functional compensation, we assessed whether the gene transcription of one was upregulated in the absence of the other via qPCR of single GCKO samples. No change in *Katnal1* mRNA expression was observed in *Katna1^GCKO/GCKO^* mice (Fig. S6A). Likewise, no change in *Katna1* mRNA expression was observed in *Katnal1^GCKO/GCKO^* mice (Fig. S6B).

### KATNA1, KATNAL1 and KATNB1 immunoprecipitate a range of cytoskeletal and vesicular transport proteins from the mouse testis

To explore the mechanisms of action and functional compensation of KATNA1 and KATNAL1 during spermatogenesis, we determined the respective interactome for KATNA1, KATNAL1 and KATNB1 within testis immunoprecipitates via MS (Table S1, Fig. S7A-C). A total of 322, 108 and 213 proteins were significantly enriched in the KATNA1, KATNAL1 and KATNB1 IPs, respectively, compared with their corresponding controls (Table S1, Fig. S7A-C). KATNA1, KATNAL1 and KATNB1 were all significantly enriched in their own IPs, validating the specificity of each IP. KATNA1 and KATNAL1 were not enriched in the IPs of each other, suggesting that they do not interact with each other to form heterohexamers (Table S1). Similarly, neither of them immunoprecipitated KATNAL2; however, KATNAL1 and KATNB1 each pulled the other down (Table S1). Consistent with the three proteins having similar functions, they shared 48 candidate interacting proteins, in addition to 102 candidates that were shared by two katanins (Fig. S7D).

PANTHER protein class and Gene Ontology analysis (Thomas et al., 2022) was conducted (Table S2, summarised in Fig. S7E-J). Consistent with their regulation of MT-based processes, in each KATNA1, KATNAL1 and KATNB1 IP, several cytoskeletal proteins were enriched (Fig. S7E-G, Table S2). Of relevance to the regulation of the Golgi and of acrosome formation, all the katanin IPs were enriched for proteins involved in vesicle-mediated transport (Fig. S7E-J, Table S2). Functional descriptions of selected cytoskeletal, membrane/vesicle-transport-related and spermatogenesis-related proteins that were significantly enriched in the KATNA1, KATNAL1 and KATNB1 IP experiments are summarised in Table S3.

## DISCUSSION

Katanin MT-severing enzymes are potent regulators of MT dynamics and organisation across eukaryotes. Herein, using a combination of single and double katanin A-subunit GCKO mice, we show the essential requirement for katanin A-subunits in mammalian male meiosis, reveal that different katanin A-subunits can functionally compensate for one another, and establish KATNA1 and KATNAL1 as regulators of spermatid differentiation (spermiogenesis). Specifically, KATNA1 and KATNAL1 collectively regulate spermatocyte metaphase and anaphase spindle structure and function, spermatocyte cytokinesis, acrosome development via the Golgi apparatus and pro-acrosomal vesicle trafficking, and sperm head shaping via manchette formation, and collectively restrain the MT bulk during male meiosis and spermiogenesis. In addition to these roles, KATNAL1 regulates sperm tail integrity, HTCA development, manchette dynamics and dissolution, and the release of sperm from the seminiferous epithelium (spermiation). Through the analysis of KATNA1, KATNAL1 and KATNB1 IPs, we reveal that these activities likely involve interactions with a diverse suite of proteins, including actin-, MT-, vesicle/membrane trafficking- and spermatogenesis-related proteins.

The finding that KATNA1 and KATNAL1, but not KATNA1 and KATNAL2, have gene redundancy in mammalian spermatogenesis is consistent with the highly similar domain structure and severing activity shared between KATNA1 and KATNAL1, but not between KATNA1 and KATNAL2 (McNally and Roll-Mecak, 2018). Interestingly, KATNAL1 fully compensates for KATNA1 germ cell loss, whereas KATNA1 only partially compensates for KATNAL1 germ cell loss. This suggests that KATNAL1 is the more potent/efficient MT-severing enzyme in the testis and/or that it has neofunctionalised to acquire additional functions. We predict that it is a combination of the two. Indeed, KATNAL1 possesses higher MT-severing activity in mouse Neuro-2a cells, and is less prone to degradation in HEK293T cells compared with KATNA1 (Hatakeyama and Hayashi, 2018). However, we also identified several different putative testis-specific KATNA1- versus KATNAL1-interacting proteins, indicating that they have at least some distinct modes of regulation and/or function. The mechanisms by which functional compensation between KATNA1 and KATNAL1 is achieved will require future exploration. The finding that it is not mediated by gene upregulation is consistent with the known mechanisms of MT-severing regulation. It has been shown that katanin A-subunits typically exist in solution in an autoinhibited monomeric state, and only assemble into active MT-severing hexamers in a MT- and ATP-dependent manner (Hartman and Vale, 1999; Nithianantham et al., 2018; Zehr et al., 2017). As such, katanin A-subunit concentration may not be a limiting step in MT severing in the testis and this may explain why an upregulation of KATNA1 or KATNAL1 content is not needed for compensation.

We identified KATNA1/KATNAL1 as essential regulators of multiple aspects of male meiosis. Although a role for katanin A-subunits in male mammalian meiosis has never previously been shown, we have previously shown that the B-subunit KATNB1 is essential for normal spindle architecture and function, and for cytokinesis in mouse spermatocytes (Dunleavy et al., 2021; O'Donnell et al., 2012). In strong support of these overlapping functions being due to KATNB1 working in partnership with KATNA1 and KATNAL1, KATNB1-KATNA1 and KATNB1-KATNAL1 complexes are present throughout mouse male meiosis, during which they localise to spindle MT fibres (Dunleavy et al., 2021). Moreover, single-cell analyses of the testis RNA transcriptome have shown that both KATNA1 and KATNAL1 are upregulated during male meiosis and spermiogenesis in mice (Ernst et al., 2019; Jung et al., 2019; summarised by Dunleavy et al., 2021). The finding that single GCKO of KATNAL1, but not of KATNA1, is sufficient to cause meiotic defects agrees with our previous data showing that KATNB1-KATNAL1 complexes are the most abundant type of katanin complex during male meiosis (Dunleavy et al., 2021).

The misalignment of metaphase chromosomes followed by uneven anaphase segregation in *Katnal1^GCKO/GCKO^* and *Katna1/al1^GCKO/GCKO^* meiosis is consistent with KATNA1/KATNAL1 regulating MT-chromosome attachment and/or regulating spindle dynamics via metaphase poleward ‘flux’ (reviewed by Rogers et al., 2005) and anaphase ‘Pacman-flux’ (reviewed by Rath and Sharp, 2011). Although future studies will need to directly test this in meiosis, katanin-mediated MT severing has been shown to directly contribute to metaphase spindle flux in HEK293 mitotic cells (Jiang et al., 2017) and to Pacman-flux in *Drosophila* mitotic cells (Zhang et al., 2007).

The role for KATNA1 and KATNAL1 in cytokinesis identified herein aligns with evidence that midbody disassembly in mitosis requires MT severing (reviewed by McNally and Roll-Mecak, 2018; additional data by Elad et al., 2011) and with our own data regarding KATNB1 function in mouse male meiosis (Dunleavy et al., 2021; O'Donnell et al., 2012). That only a subset of germ cells fails cytokinesis in the absence of KATNB1 or KATNA1 and KATNAL1 suggests that other MT-severing enzymes must also be involved in this process. Indeed, we have recently shown that the related MT-severing enzyme, spastin, also contributes to cytokinesis during male meiosis (Cheers et al., 2023).

Our data establish KATNA1 and KATNAL1 as regulators of Golgi organisation and pro-acrosomal vesicle trafficking and fusion during acrosome biogenesis. This contributes to an emerging body of evidence that MT-severing enzymes regulate vesicle-based transport in spermiogenesis, as we have recently shown that spastin and KATNB1 are also essential for pro-acrosomal vesicle transport and fusion events (Cheers et al., 2023; Dunleavy et al., 2021). The contribution of KATNA1/KATNAL1 to these processes may be as simple as a role in pruning the MT tracks along which Golgi membranes are spatially positioned and pro-acrosomal vesicles are delivered. However, the identification in our katanin IPs of multiple proteins involved in Golgi organisation and vesicle trafficking suggests a more direct role and raises the possibility that MT severing can modulate cargo-MT interactions.

We have previously shown that KATNAL2 is essential for sperm tail development via basal body-plasma membrane docking and the initiation of spermatid axoneme extension (Dunleavy et al., 2017). Here, we show that KATNAL1 is also required for formation of stable sperm flagella and for HTCA formation/integrity. The precise mechanism of KATNAL1 contribution to these processes remains an outstanding question. One possibility is that KATNAL1-mediated MT severing directly regulates MT axoneme stability and HTCA fortification. Insufficient integrity of these structures could also be consistent with mis-regulated cargo trafficking. Of note, and consistent with the co-incident manchette defects in KATNAL1 loss, sperm tail components including the ODFs and proteins required to strengthen the HTCA appear to be delivered via the MTs of the manchette, and abnormal sperm tail and manchette phenotypes often occur in parallel (reviewed by Lehti and Sironen, 2016). It is thus possible that the abnormal manchette phenotypes in *Katnal1^GCKO/GCKO^* mice underpin at least some of the sperm tail defects.

The manchette phenotypes identified in the *Katnal1^GCKO/GCKO^* mice establish roles for KATNAL1 in manchette migration down the sperm nucleus, which requires ‘unzipping’ of rod-like manchette-nuclear linkers, and in controlling manchette MT length as well as in manchette MT disassembly. These functions are consistent with a direct MT-severing mechanism. Strikingly, the *Katnal1^GCKO/GCKO^* mouse manchette phenotypes mirror not only those in KATNB1 loss-of-function mice (Dunleavy et al., 2021; O'Donnell et al., 2012), but also those seen upon KATNAL2 loss (Dunleavy et al., 2017). These identical yet non-redundant KATNAL1 and KATNAL2 phenotypes suggest that the two proteins execute similar functions in the manchette but sever different MT subpopulations. This is consistent with our proteomics data, demonstrating that KATNAL2 and KATNAL1 do not immunoprecipitate one another.

Similar to the established roles of Sertoli cell KATNAL1 in maintaining round spermatid-Sertoli cell adhesion (Smith et al., 2012), we show that germ cell KATNAL1 is involved in spermatid retention and elongated spermatid release via spermiation. Our data are consistent with KATNAL1 severing the cytoskeletal connections that tether elongating spermatids to Sertoli cells. The precise cytoskeletal targets of this action need to be identified but, of note, our IP data suggest that katanins interact with multiple ARP2/3 complex proteins. The ARP2/3 complex appears to facilitate spermiation, albeit via the disassembly of the Sertoli cell actin cytoskeleton (O'Donnell et al., 2011). Of note, spermiation defects in *Katnal2* mutant mice (Dunleavy et al., 2017) and spermatid adhesion and spermiation defects seen in *Katnb1* mutant mice (Dunleavy et al., 2021; O'Donnell et al., 2012) phenocopy those seen in *Katnal1^GCKO/GCKO^* mice. This suggests that KATNAL1-KATNB1 complexes along with KATNAL2-KATNB1 complexes facilitate spermatid release.

Finally, we defined the testis interactome for KATNA1, KATNAL1 and KATNB1. Although a katanin interactome has previously been defined in HeLa cells (Cheung et al., 2016), this is the first *in vivo* tissue-wide interactome for each protein. A number of protein classes were identified that we predict are related to the activation/inhibition of katanin function (e.g. metabolite interconversion enzymes). Of proteins that we predict are involved in testis-specific functions of the katanin enzymes, cytoskeletal and vesicle-transport related proteins emerged as key protein classes. Of the MT-related proteins identified, multiple proteins are involved in the regulation and activity of dynein MT motor proteins. Consistent with the phenotypes of our *Katnb1* (Dunleavy et al., 2021; O'Donnell et al., 2012), *Katnal1* and *Katna1/al1* mutant mice, the MT-related proteins identified also included those with established functions in the mitotic and/or meiotic spindle (e.g. NUDC and MAPRE1/EB1), intraflagellar transport and ciliogenesis (ARL3), and axoneme assembly (DNAAF4), and several have previously been implicated in spermatogenesis and male fertility (e.g. ARL3, DNAAF4 and MAPRE1/EB1). Notably, our data suggest that KATNA1, KATNAL1 and KATNB1 bind multiple regulators of actin network organisation and actin polymerisation/dynamics. These include ARP2/3 complex components, proteins which previous data strongly suggest play key roles in blood-testis barrier integrity, spermiogenesis and spermiation (Li et al., 2015; Lie et al., 2010; Liu et al., 2018; O'Donnell et al., 2011). This potential connection between katanin proteins and the actin cytoskeleton in the testis is consistent with data showing that the *Arabidopsis* katanin A-subunit KATANIN 1 regulates the actin cytoskeleton (Takáč et al., 2017) and that cortical localisation of the *Drosophila* KATNA1 orthologue, Kat60, is actin dependent (Zhang et al., 2011).

Our katanin testis interactome data also suggest that KATNA1 and KATNB1 bind several Rab GTPases, a family that regulates multiple aspects of vesicle transport (vesicle budding, actin/MT-mediated transport and fusion). In spermatogenesis, there is increasing evidence that Rab and Rab-like proteins mediate pro-acrosomal vesicle transport, in addition to intraflagellar transport and sperm tail development (Blacque et al., 2018; Lo et al., 2012; Ramalho-Santos et al., 2001; Shan and Sun, 2021). Other transport proteins identified in the katanin testis interactome include those related to endoplasmic reticulum to cis-Golgi transport, intra-Golgi transport, clathrin-mediated vesicle disassembly and endocytosis. Consistent with the *Katna1/al1^GCKO/GCKO^* mouse acrosome defects, the KATNAL1 IP was enriched in ACRBP, a protein essential for acrosome granule formation and maintenance of acrosome integrity, and, consistent with the Golgi defects, a number of proteins were identified with roles in Golgi organisation [e.g. Rab2a/b, Rab6a/b, VCP and LMAN1]. Future studies will need to validate and define the precise nature of the interactions identified in the katanin testis interactome; however, collectively, they paint a picture of katanin action involving a complex crosstalk between different cytoskeletal networks, notably between the actin and MT networks, and suggest a direct role for katanins in regulating the interaction of dynein- and vesicle-based cargo machinery with MTs.

**Fig. 7.**
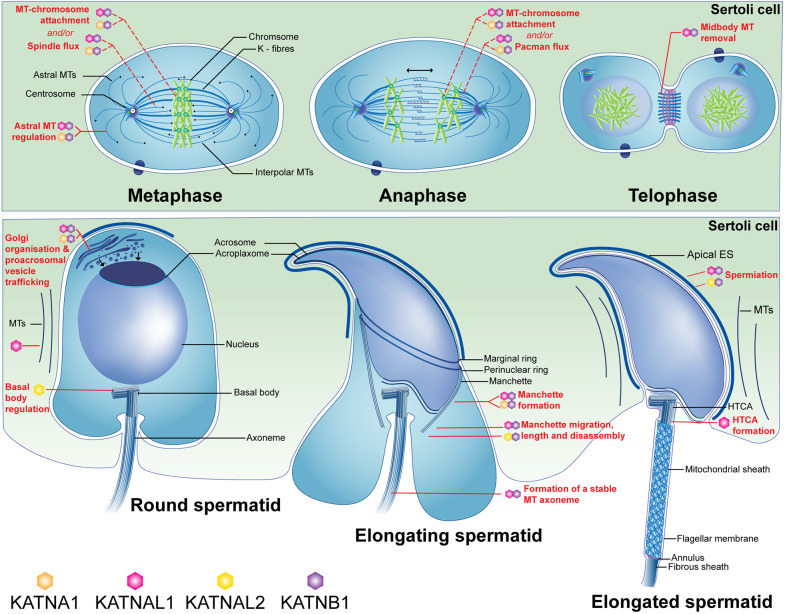
**Proposed model of katanin regulation of spermatogenesis.** KATNB1-independent and -dependent functions of each katanin A-subunit during male germ cell development based on data defined herein and previously for KATNAL1 Sertoli cell function (Smith et al., 2012), KATNAL2 (Dunleavy et al., 2017) and KATNB1 (Dunleavy et al., 2021; O'Donnell et al., 2012). A shared line indicates two complexes have redundant/compensatory function in the process indicated. ES, ectoplasmic specialisation. Schematic was prepared by the authors using Adobe Illustrator.

This study establishes KATNA1 and KATNAL1 as key players in male germ cell MT regulation. The data defined herein, taken together with our previous data on the roles of KATNB1 (Dunleavy et al., 2021; O'Donnell et al., 2012), KATNAL2 (Dunleavy et al., 2017) and Sertoli cell KATNAL1 (Smith et al., 2012), allow us to define a model of the complete katanin ‘toolbox’ during mammalian spermatogenesis (Fig. 7). These data unequivocally show that the emergence of multiple katanin A-subunits in higher eukaryotes has allowed not only for functional sub-specialisation and thus customised cutting of discrete MT subpopulations, but also for the protection of katanin function in crucial processes such as meiosis through gene redundancy.

## MATERIALS AND METHODS

### Mouse model production

All animal procedures were approved by the Monash University School of Biological Sciences Animal Experimentation Ethics Committee and the University of Melbourne Ethics committee and conducted in accordance with National Health and Medical Research Council (NHMRC) Guidelines on Ethics in Animal Experimentation.

To the generate *Katna1^Flox/Flox^* mice, *Katna1* knockout-first conditional-ready mice (*Katna1^KO/WT^*) were generated through the Monash University Node of the Australian Phenomics Network using a EUCOMM *Katna1* knockout-first conditional-ready embryonic stem cell clone (EPD0586_1_B10) as described previously (Cotton et al., 2015). To disrupt the *Katna1* gene, the *FRT*-*lacZ*-*loxP*-*Neo*-*FRT*-*loxP*-*Katna1*-exons6-7-*loxP* cassette was inserted into intron 5 of the *Katna1* gene (Fig. S1C). To generate conditional *Katna1* knockout mice (*Katna1^Flox/Flox^*), mice heterozygous for the knockout-first allele (*Katna1^KO/WT^*) were then crossed with a transgenic line carrying the *Flp* recombinase gene. This resulted in removal of the *FRT*-flanked gene-trap cassette, exposing the floxed *Katnal1* exons 6 and 7 and reverting mice to a WT phenotype. *Katna1^GCKO/GCKO^* were then generated using a two-step breeding strategy: (1) *Katna1^Flox/Flox^* females were mated with a *Stra-8-cre* transgenic line to produce *Katna1^WT/Flox,Stra8-cre+^* males; then (2) *Katna1^WT/Flox,Stra8-cre+^* males were mated with *Katna1^Flox/Flox^* females to produce *Katna1^Flox/Flox,Stra8-cre+^* progeny (*Katna1^GCKO/GCKO^*). In these mice, gene disruption relied on Cre-lox recombination-mediated excision of *Katna1* exons 6 and 7 (ENSMUSE00001254109, ENSMUSE00001247569), and the *Stra8-Cre* used was expressed from late spermatogonia onwards (Sadate–Ngatchou et al., 2008).

*Katnal1^Flox/Flox^* mice, wherein exon 3 (ENSMUSE00001360953) of the *Katnal1* gene is flanked by *loxP* sites, were generated by the Monash Genome Modification Platform using CRISPR/Cas9 technology. Guide RNA target sites flanking the target exon (5′ end of the exon, 5′-TCAATGTCTGAGGTGGATAA-3′; 3′ end of the exon, 5′-CAAAGAAGTCTTTTAGACCC-3′) were identified using the UCSC Genome Browser (https://genome.ucsc.edu/). A single-stranded DNA (ssDNA) homology-directed repair template was generated from a *Katnal1* conditional knockout targeting construct using the Guide-it Long ssDNA Strandase Kit (Takara, 632645). The following oligonucleotides were used to generate a PCR product from which the ssDNA was generated: 5′-GATGCTTAGCACTGGCTGG-3′ and 5′-GAACGTGTGCTATTTGGTCTTTAAG-3′. The CRISPR/Cas9 ribonucleoprotein complex and ssDNA homology repair templates were microinjected into C57BL/6J zygotes at the pronuclei stage and transferred into the uterus of pseudo-pregnant females. *Katnal1^GCKO/GCKO^* mice were generated by intercrossing *Katnal1^Flox/Flox^* mice with a *Stra8-cre* transgenic line as described above. *Katnal2^Flox/Flox^* mice were generated as previously described (Dunleavy et al., 2017). *Katna1/al1^Flox/Flox^* and *Katna1/al2^Flox/Flox^* mice were generated by breeding *Katna1^Flox/Flox^* mice with either *Katnal1^Flox/Flox^* or *Katnal2^Flox/Flox^* mice, respectively. Double germ cell deletion was then achieved using by intercrossing double-floxed mice with a *Stra8-cre* transgenic line as described above.

All mice were maintained on a C57BL/6 background. To control for background strain effects on phenotype, *Katna1^Flox/Flox^*, *Katnal1^Flox/Flox^*, *Katna1/al1^Flox/Flox^* and *Katna1/al2^Flox/Flox^* mice were used as controls for *Katna1^GCKO/GCKO^*, *Katnal1^GCKO/GCKO^*, *Katna1/al1^GCKO/GCKO^* and *Katna1/al2^GCKO/GCKO^* mice, respectively. Mouse genotypes were determined from tail biopsies using real-time PCR with specific probes designed for each allele (Transnetyx, Cordova, TN, USA). Efficiency of exon excision in *Katna1^GCKO/GCKO^*, *Katnal1^GCKO/GCKO^*, *Katna1/al1^GCKO/GCKO^* and *Katna1/al2^GCKO/GCKO^* mice was determined by qPCR of isolated germ cells as described below. Protein levels were determined by western blotting of germ cell and testis lysates as described below.

### qPCR

Purified germ cells were prepared via the STAPUT method of gravity sedimentation as detailed in Dunleavy et al. (2019). Total RNA was then extracted from purified germ cells using Trizol (Life Technologies) and reverse-transcribed into cDNA using SuperScript III Reverse Transcriptase (Life Technologies). Primers spanning *Katna1* exons 6 and 7 (forward, 5′-TCAGGAAGCAGTGGTGTTACC-3′, and reverse, 5′**-**AAGGGTCTTTCCAGTGCCAG-3′), *Katnal1* exons 3 and 4 (forward, 5′-CCGTGTCTTGCCGAGATGAA-3′, and reverse, 5′-GCGATTTGGACGCCTGATTT-3′), and *Katnal2* exons 2 and 3 (forward 5′-AAGGCTTTGGAGGAGGAGAC-3′, and reverse, 5′**-**GGGGCCTTCTTAACCACTTT-3′) were used to verify gene deletion in the corresponding mouse models. All qPCRs were performed using SYBR Select Master Mix (Applied Biosystems). Each reaction was performed in triplicate using the Agilent Mx3000P qPCR system or an Applied Biosystems QuantStudio 3 Real-Time PCR System with the parameters of 50°C for 2 min, 95°C for 10 min, followed by 40 cycles of 95°C for 15 s and 60°C for 1 min. *Ppia* was amplified at the same time as an internal control (using the forward primer 5′-CGTCTCCTTCGAGCTGTTT-3′ and reverse primer 5′-CCCTGGCACATGAATCCT-3′), and all results were normalised to *Ppia* expression. Differential expression was analysed using the 2^ΔΔCT^ method (Livak and Schmittgen, 2001).

### Male fertility characterisation

Male fertility of all mouse models was characterised as per Houston et al. (2020). Male fertility was assessed by mating 10- to 12-week-old males with WT females (≥6 weeks of age). Copulatory plugs were monitored to confirm successful mating and the number of pups born per copulatory plug was recorded. Each male was test mated with two to three females and pup numbers were averaged. Testis DSP and total epidydimal sperm content were determined using the Triton X-100 solubilisation method (Cotton et al., 2006) (*n*≥3/genotype) as modified in Dunleavy et al. (2021). Briefly, this involved quantification of the number of elongated spermatids/sperm per testis and epididymis using a haemocytometer. Testis DSP rates were then calculated by dividing the number of sperm per testis by 4.84 (time divisor, i.e. the number of days detergent-resistant elongated spermatids reside in the mouse testis; Russell et al., 1993). To assess sperm motility, sperm were backflushed as per Baker et al. (2014) from cauda epididymides and motility was assessed using a Computer-Assisted Sperm Analysis system (MouseTraxx, Hamilton Thorne), as described previously (Gibbs et al., 2011).

### Histology

Testes and epididymal tissue were fixed in Bouin's fixative and histology was assessed using standard methods. Male reproductive tract histology was visualised using PAS and Haematoxylin staining. Air-dried caudal epididymal sperm smears were visualised using Haematoxylin and Eosin staining (*n*≥3/genotype), and, for each mouse, the percentage of morphologically abnormal sperm was quantified by assessment of ≥100 randomly selected sperm (*n*≥3 mice/genotype). The proportions of sperm with abnormal head shapes, abnormal head position relative to the tail, decapitated heads, coiled mid-pieces and mitochondrial sheath defects were also quantified and statistical analysis conducted as described below. Germ cell apoptosis was evaluated by immunostaining for cleaved caspase 3 and 7 as per O'Bryan et al. (2013) and as described below. The numbers of caspase-positive cells in a minimum of 50 randomly selected seminiferous tubules per mouse were counted and statistical analysis conducted as described below and as per Dunleavy et al. (2021) (*n*≥3 mice/genotype). Metaphase chromosome misalignment in spermatocytes was evaluated by immunostaining for α-tubulin as a marker of metaphase spindle MTs as described below. The percentage of metaphase spermatocytes with misaligned chromosomes was quantified by assessment of ≥50 metaphase spermatocytes per mouse (*n*≥3 mice/genotype). Binucleated spermatids were quantified by scoring all spermatids in a minimum of ten stage VIII PAS-stained seminiferous tubule cross-sections per mouse as being either single nucleated or binucleated (*n*≥3 mice/genotype). Acrosome abnormalities were quantified by scoring all spermatids in a minimum of three stage XI PAS-stained seminiferous tubule cross-sections per mouse as having either normal or abnormal acrosome morphology (*n*=4 mice/genotype). Statistical analysis was conducted as detailed below.

### TEM

For analysis of seminiferous tubule ultrastructure, partially decapsulated testes and cauda epididymides were fixed with 5% glutaraldehyde/4% paraformaldehyde/0.02% picric acid in 0.1 M sodium cacodylate buffer, pH 7.4, as per Dunleavy et al. (2021). Samples were *en bloc* stained with 2% uranyl acetate and all samples were embedded into Epon resin using standard procedures. Ultrathin sections were cut on a Leica EM UC7 ultramicrotome and placed on copper 100×100 square grids (ProSciTech). Sections were analysed using a FEI Talos L120CI TEM in the Ian Holmes Imaging Centre (The University of Melbourne, Parkville, Australia).

### Western blotting

Immunoblotting was conducted as previously described (Dunleavy et al., 2019). Briefly, proteins were extracted from STAPUT-purified germ cells (Dunleavy et al., 2019) using RIPA buffer (50 mM Tris-HCl; 1% NP-40; 0.1% SDS; 0.5% sodium deoxycholate (w/v); 0.9% NaCl (w/v); 5 mM EDTA, pH 7.4) with protease inhibitor cocktail (Calbiochem, USA). The DC Protein Assay Kit II (Bio-Rad, 5000112) was used to perform Lowry protein concentration assays. Proteins were separated by polyacrylamide gel electrophoresis on a 10% SDS gel (∼25 μg of protein was loaded per lane), transferred to PVDF membranes and probed using primary antibodies. To detect KATNAL1 via western blotting, our in-house anti-KATNAL1 antibody was used (as described previously; Dunleavy et al., 2021), and actin (A2066, Sigma-Aldrich, 0.67 μg ml^−1^) was used as a loading control. Bound antibodies were detected using a goat anti-rabbit immunoglobulin/horseradish peroxidase (P0448, Dako, 1:10,000) secondary antibody with Clarity ECL substrate (Bio-Rad). Images were captured using a ChemiDoc MP Imaging System (Bio-Rad). The molecular masses of detected proteins were estimated using a PageRuler Plus Prestained Protein Ladder (Thermo Fisher Scientific) and the Image Lab 6.0 Molecular Weight Analysis Tool (BioRad).

### Fluorescence immunohistochemistry and imaging

To assess KATNA1 testis localisation via immunofluorescence labelling, 5 µm-thick cryostat testis sections were fixed in 1:1 methanol-acetone and permeabilised in 0.2% Triton X-100 in PBS as per Gibbs et al. (2010). To assess testis localisation of other proteins via immunofluorescence labelling, Bouin's fixed testes were processed into paraffin and 5 µm-thick sections prepared using standard methods. Paraffin sections were dewaxed and heat-mediated antigen retrieval was conducted by microwaving sections in 10 mM citrate buffer (pH 6) for 16 min as per Jamsai et al. (2008). For all testis immunofluorescence labelling, non-specific protein binding was minimised by incubation with CAS-block (Invitrogen) for 30 min and sections were then incubated with primary antibodies diluted in DAKO Antibody Diluent overnight at 4°C. The primary antibodies used included our in-house anti-KATNA1 and anti-KATNAL1 antibodies (as described previously; Dunleavy et al., 2021), and an anti-TUBB3 antibody (Sigma-Aldrich, SAB3300047, 0.15 μg ml^−1^). Sections were then incubated with the appropriate Alexa Fluor-conjugated secondary antibodies diluted 1:500 for 1 h at room temperature. The secondary antibodies included Alexa Fluor 488 donkey anti-mouse IgG (A212062, Invitrogen), Alexa Fluor 488 donkey anti-rabbit IgG (A21206, Invitrogen) and Alexa Fluor 555 donkey anti-rabbit IgG (A31572, Invitrogen). DNA was then visualised using 1 µg ml^−1^ 4′,6-diamidino-2-phenylindole (DAPI, Invitrogen) or 4 µM TO-PRO-3 iodide (TOPRO, Thermo Fisher Scientific). Acrosomes were visualised using 0.5 µg ml^−1^ lectin peanut agglutinin (PNA), Alexa Fluor 488 conjugate (L21409, Life Technologies). Immunofluorescence images of testis sections were collected using a Leica TCS SP8 confocal microscope (Leica Microsystems) at Monash Micro Imaging (Monash University) and the University of Melbourne Biological Optical Microscopy Unit (University of Melbourne, Parkville, Australia). A 63×/1.40 HC PL APO CS2 oil immersion objective was used to take all immunofluorescence images.

To assess SUN5 localisation in cauda epididymal sperm, sperm were washed in PBS and airdried onto slides. Sperm were then fixed in 4% paraformaldehyde for 10 min. Next, sperm were permeabilised, blocked for non-specific protein binding and immunofluorescently labelled as above using a primary antibody against SUN5 (ProteinTech, 17495-1-A,P 0.8 µg ml^−1^) and an Alexa Fluor 488 donkey anti-rabbit antibody (A21206, Invitrogen, 1:500). DNA was then visualised using 1 µg ml^−1^ DAPI. Fluorescence images of immunolabelled sperm were collected using cellSens Imaging Software (Olympus) on an Olympus BX53 light microscope equipped with an Olympus DP80 camera.

### Chromogenic immunohistochemistry and imaging

To assess testis localisation of proteins via chromogenic immunohistochemistry, 5 µm-thick Bouin's-fixed paraffin-embedded testes sections were prepared and heat-mediated antigen retrieval was conducted as above. Endogenous peroxidase activity was then blocked via incubation with 5% H_2_O_2_ for 5 min and non-specific protein binding was minimised by incubation with CAS-block (Invitrogen) for 30 min. Sections were then incubated with primary antibodies diluted in DAKO Antibody Diluent overnight at 4°C. The primary antibodies used included those against acetylated-tubulin (T6793, Sigma-Aldrich, RRID: Ab_477585, ascites fluid, 1:10,000), α-tubulin (T5168, Sigma-Aldrich, ascites fluid, 1:50,000), cleaved caspase 3 (9664, Cell Signaling Technology, 0.5 μg ml^−1^) and cleaved caspase 7 (9491, Cell Signaling Technology, 0.23 μg ml^−1^). Sections were incubated with DAKO EnVision+ Dual Link System-HRP for 30 min at room temperature and the Liquid DAB+ Substrate Chromogen System (DAKO, K3468) was then applied as per the manufacturer's instructions. Nuclei were counterstained using Mayer's Haematoxylin (Amber Scientific), followed by Scott's tap water (Amber Scientific). Bright-field images were collected using cellSens Imaging Software (Olympus) on an Olympus BX53 light microscope equipped with an Olympus DP80 camera.

### IP-MS

For IP assays, our in-house KATNA1, KATNAL1 and KATNB1 antibodies were used (Dunleavy et al., 2021). WT mouse testes (*n*=3 per IP assay) were lysed in 1% NP-40 (PBS) supplemented with protease inhibitor cocktail (Calbiochem, 539134). KATNA1, KATNAL1 and KATNB1 and their binding partners were then co-immunoprecipitated using the Pierce Co-immunoprecipitation Kit (Thermo Scientific, 26149) as per the manufacturer's instructions, in combination with either our anti-KATNA1 (10 µg per IP column), anti-KATNAL1 (5 µg) or anti-KATNB1 (5 µg) inhouse antibody. Briefly, to reduce non-specific binding, testis protein lysates were first pre-cleared by incubation with 400 μl of Pierce control agarose resin slurry for 0.5-1 h at 4°C with gentle rocking. For each pre-cleared testis lysate, 5 mg was then loaded into two IP columns, one that contained the target antibody covalently coupled to the amine-reactive Pierce AminoLink Plus Coupling Resin and one that contained the target antibody and a non-amine-reactive Pierce Control Agarose Resin (negative control), and incubated overnight at 4°C. Lysate flowthrough was removed, columns were washed, and bound protein was eluted and collected as per the manufacturer's instructions. The eluted proteins were neutralised with 1.5 M Tris-HCl (pH 8.8), reduced with Tris(2-carboxyethyl)phosphine (TCEP), then digested by incubation with trypsin overnight at 37°C. Peptide samples were freeze dried and resuspended in 2% acetonitrile (ACN)/0.1% formic acid (FA) (diluted in MilliQ water) before concentrated FA was added to adjust the pH to <3. Samples were purified using reverse-phase chromatography C18 stage tips, which aim to desalt and fractionate in-solution peptides at an acidic pH. The tips were initially activated with 100% methanol and equilibrated with 0.1% FA, before samples were loaded into them and desalted with 0.1% FA. Peptides were eluted with 80% ACN/0.1% FA, freeze dried, resuspended in 2% ACN/0.1% FA and sonicated for 15 min in a water bath.

The purified peptide samples were analysed via nano-liquid chromatography coupled to tandem mass spectrometry (LC-MS/MS). Briefly, the KATNA1 and KATNB1 IP samples were analysed at the Monash Proteomics and Metabolomics Facility (Monash University) using an Orbitrap Fusion Tribrid mass spectrometer (Thermo Fisher Scientific) and a Q-Exactive Hybrid Quadrupole-Orbitrap mass spectrometer (Thermo Fisher Scientific), respectively. Both instruments were coupled to an UltiMate 3000 RSLCnano system (Thermo Fisher Scientific) equipped with an Acclaim PepMap RSLC C18 nanoViper Analytical Column (100 Å, 75 μm×50 cm, 2 μm; Thermo Fisher Scientific) and an Acclaim PepMap 100 C18 HPLC nanoViper Trap Column (100 Å, 100 μm×2 cm, 5 μm; Thermo Fisher Scientific). The tryptic peptides were separated by increasing concentrations of 80% ACN/0.1% FA at a flow rate of 250 nl/min for 128 min and 158 min, respectively. Both mass spectrometers were operated in data-dependent acquisition mode to automatically switch between full-scan MS and MS/MS acquisition. Each survey full scan (mass-to-charge ratio or m/z 375–1800) in the Orbitrap Fusion Tribrid acquisitions was acquired in the Orbitrap with 120,000 resolution (at m/z 200) after accumulating ions to a normalised automatic gain control (AGC) target of 250% with a maximum injection time of 54 ms. Dynamic exclusion was set to 15 s. Adhering to a 2 s cycle time, the most intense multiply charged ions (z≥2) were sequentially isolated and fragmented in the collision cell by higher-energy collisional dissociation and MS2 scans were acquired with a fixed injection time of 54 ms, 30,000 resolution and a normalised AGC target of 400%. Very similar parameters were used for the Q-Exactive Hybrid Quadrupole – Orbitrap acquisitions.

The KATNAL1 IP-purified peptide samples were analysed at the University of Melbourne Mass Spectrometry and Proteomics Facility using an Orbitrap Eclipse Tribrid Mass Spectrometer (Thermo Fisher Scientific) equipped with a nano ESI interface coupled to an Ultimate 3000 nano HPLC (Thermo Fisher Scientific). The LC system was equipped with an Acclaim Pepmap 100 C18 HPLC nanoViper Trap Column (100 Å, 75 μm×2 cm, 3 μm; Thermo Fisher Scientific) and an analytical column as above. The enrichment column was injected with the tryptic peptides (1 µl, 100 ng) at an isocratic flow of 5 μl/min of 2% v/v CH_3_CN containing 0.05% v/v trifluoroacetic acid for 6 min, applied before the enrichment column was switched in-line with the analytical column. The eluents were 0.1% v/v FA (solvent A) in H_2_O and 100% v/v CH_3_CN in 0.1% v/v FA (solvent B). The gradient was at 300 nl min^−1^ from: (1) 0-6 min, 3% solvent B; (ii) 6-35 min, 3-23% solvent B; (3) 35-45 min, 23-40% solvent B; (4) 45-50 min, 40-80% solvent B; (5) 50-55 min, 80-80% solvent B; (6) 55-55.1 min, 80-3% solvent B; and (7) 55.1-65 min, 3-3% solvent B. The Eclipse Orbitrap mass spectrometer was operated in the data-dependent mode, wherein full MS1 spectra were acquired in a positive mode over the range of m/z 375-1500, with spray voltage at 1.9 kV, source temperature at 275°C, MS1 at 120,000 resolution, normalised AGC target of 100% and maximum injection time of 22 ms. The top 3 s method was used and selecting peptide ions with charge states of ≥2-7 and intensity thresholds of ≥5E4 were isolated for MS/MS. The isolation window was set at 1.6 m/z, and precursors were fragmented using higher-energy C-trap dissociation at a normalised collision energy of 30%, a resolution of 15,000, a normalised AGC target of 100% and automated injection time.

The raw MS data were analysed with the MaxQuant software suite for the identification and quantification of peptides/proteins from the Mouse SwissProt database. Fixed modifications of carbamidomethylation of cysteine as well as variable oxidation of methionine and protein N-terminal acetylation were considered. Trypsin/P was set as the protease with a maximum of two missed cleavages. Statistical analysis using a two-tailed unpaired *t*-test was conducted with the Perseus software considering protein and peptide-spectrum match (PSM) false discovery rates (FDRs) both set at <0.01. Significance was defined as *P*<0.05. For each IP assay, the majority UniProt ID(s) for each significantly enriched protein group were then analysed using PANTHER Gene Ontology and protein classification (Thomas et al., 2022). All MS proteomics data have been deposited to the ProteomeXchange Consortium via the PRIDE partner repository with the dataset identifier PXD038404 (Perez-Riverol et al., 2021).

### Statistics

Statistical analysis of germ cell apoptosis data was conducted in R version 3.5.1. Generalised linear mixed models were used to compare the number of caspase-positive cells per tubule across genotypes, with animal ID included as a random effect to account for repeated measures per individual. For each model, Akaike information criteria (AIC) estimates were used to select the most appropriate error distribution and link function (i.e. Poisson, negative binomial, zero-inflated Poisson, zero-inflated negative binomial) using the glmer function (lme4 package; Bates et al., 2015) and glmmTMB function (glmmTMB package; Brooks et al., 2017). For all models, a zero-inflated negative binomial distribution (fitted with glmmTMB, using the ziformula argument) was selected as the most appropriate error distribution and link function (i.e. had the lowest AIC score).

All other statistical analysis, other than for the proteomics (described above), was conducted in GraphPad Prism 9.0. The statistical significance of differences between two groups was determined with an unpaired two-tailed Student’s *t*-test. Significance was defined as *P*<0.05.

## Supplementary Material

10.1242/develop.201956_sup1 Supplementary information

Table S1. Significantly enriched proteins identified in KATNA1, KATNAL1 and KATNB1 testis co-immunoprecipitation experiments with subsequent mass spectrometry analysis.

Table S2. PANTHER analysis of proteins identified by mass spectrometry as significantly enriched in either KATNA1, KATNAL1 or KATNB1 testis co-immunoprecipitates.

Table S3. Functional descriptions of selected KATNA1 (A1), KATNAL1 (AL1) and KATNB1 (B1) candidate testis binding proteins of interest.
