# Supplementary material for: The katanin A-subunits KATNA1 and KATNAL1 act co-operatively in mammalian meiosis and spermiogenesis to achieve male fertility
Source: Development. 2023 Nov 13;150(22):dev201956. doi: 10.1242/dev.201956 (PMC10690054; doi:10.1242/dev.201956)
Supplement: Supplementary information [file develop-150-201956-s1.pdf]

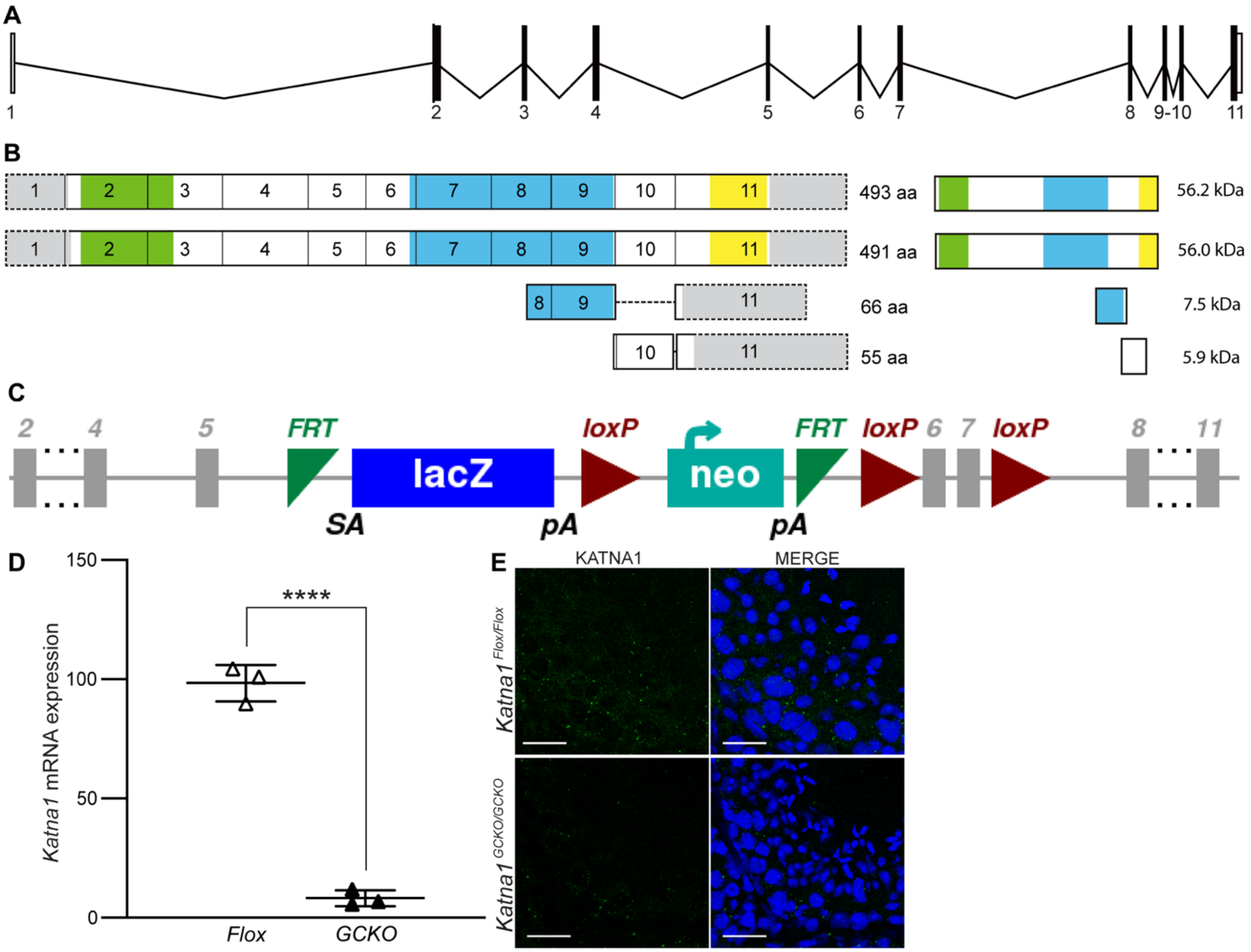

Fig. S1. Generation of the *Katna1*$^{GCKO/GCKO}$ mouse model

Katnal1 gene (A) and transcript structure (B). The MT interacting and transport (MIT) domain, AAA ATPase domain and VPS4 domain are shown in green, blue and yellow respectively. Untranslated regions are shown in grey. (C) The *Katna1* KO-first conditional ready cassette. The FRT-lacZ-loxP-Neo-FRT-loxP-*Katna1* exons 6- 7-loxP cassette was inserted into *Katna1* intron 5. (D) qPCR analysis of *Katna1* transcript levels in *Katna1*$^{GCKO/GCKO}$ (black triangles) relative to *Katna1*$^{Flox/Flox}$ (white triangles) isolated spermatocytes (n=3/genotype). Data are normalised to *Ppia,* and lines represent mean ±s.d. ****P<0.0001. (E) *Katna1*$^{Flox/Flox}$ and *Katna1*$^{GCKO/GCKO}$ testis sections immunolabelled for KATNA1 (green). Nuclei are counterstained with DAPI (blue). Scale bars in F = 20 µm.

Development • Supplementary information

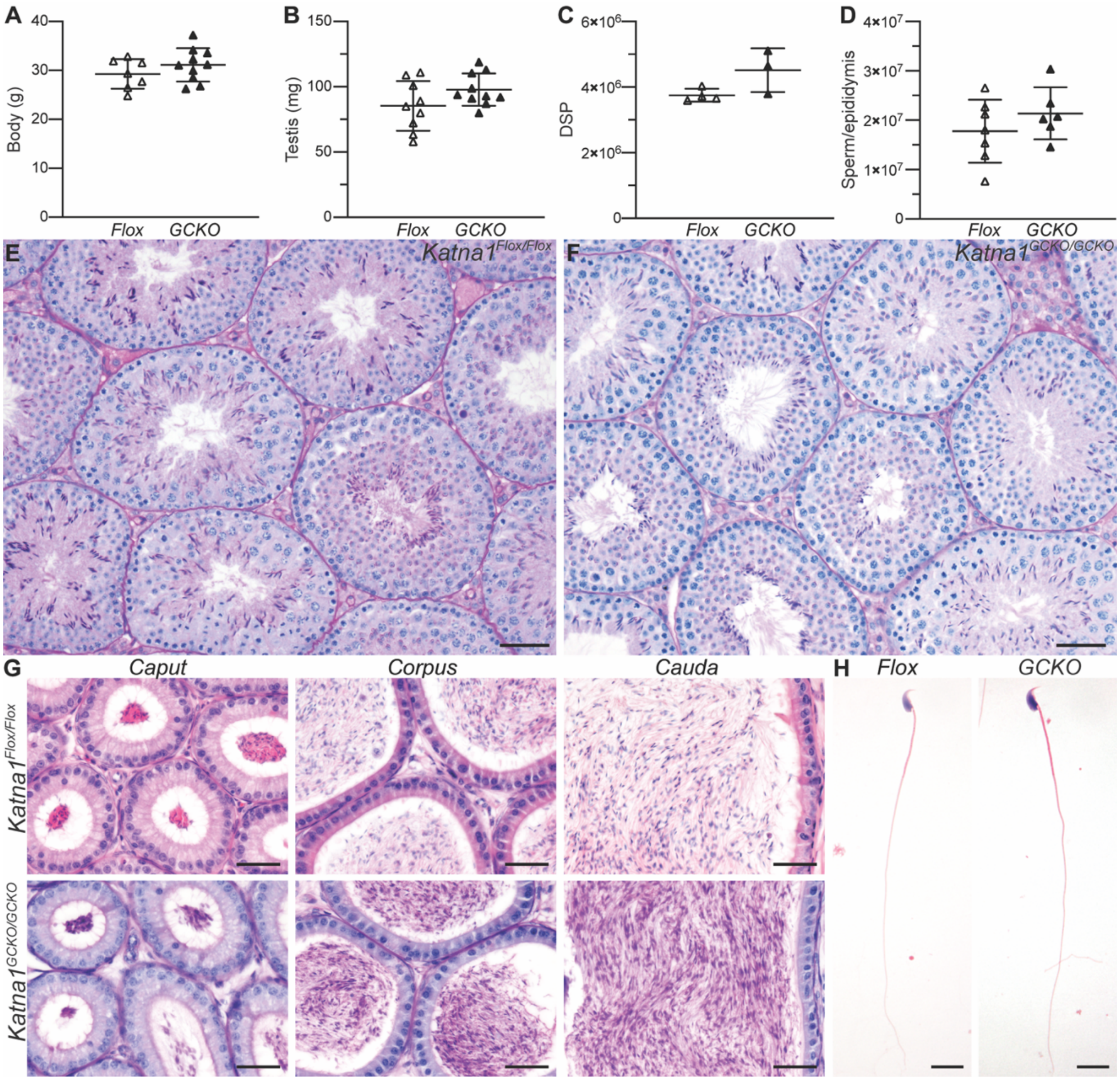

Fig. S2. KATNA1 is dispensable for male meiosis and haploid germ cell development

Body weight (A), testis weight (B), testis DSP (C), and epididymal sperm content (D) in *Katna1*$^{GCKO/GCKO}$ mice (black triangles) and *Katna1*$^{Flox/Flox}$ controls (white triangles) (n≥3/genotype). Error bars represent mean±s.d. PAS-stained testis (E-F) and epididymis (G) sections, and hematoxylin and eosin-stained cauda epididymal sperm (H) from *Katna1*$^{GCKO/GCKO}$ and *Katna1*$^{Flox/Flox}$ mice. Scale bars in E-F = 50 µm, G = 40 µm and H = 10 µm.

Development • Supplementary information

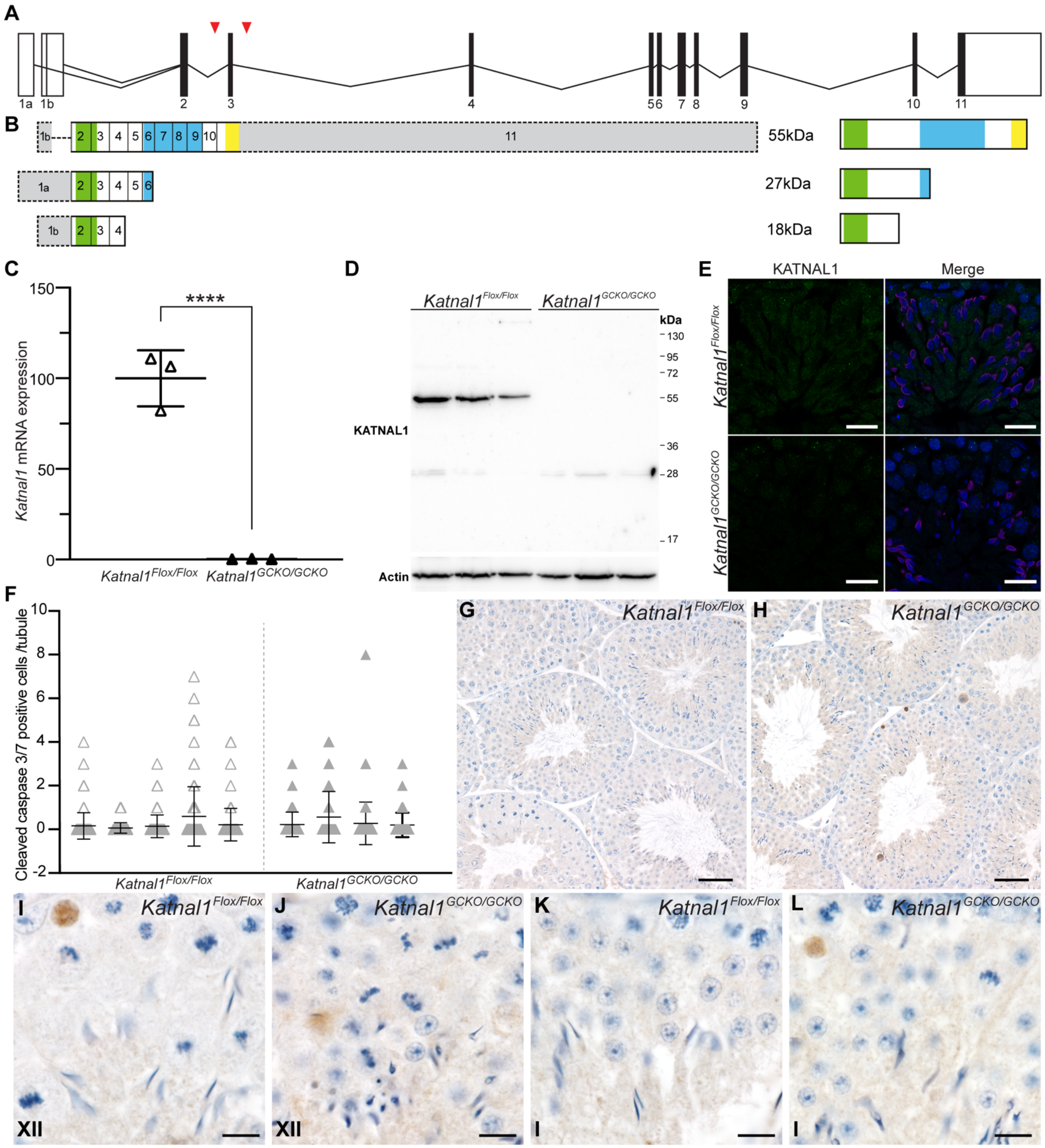
A
1a
1b
2
3
4
5
6
7
8
9
10
11
B
55kDa
27kDa
18kDa
C
Katnal1 mRNA expression
****
Katnal1Flox/Flox
Katnal1GCKO/GCKO
D
KATNAL1
Actin
kDa
130
95
72
55
36
28
17
E
KATNAL1
Merge
F
Cleaved caspase 3/7 positive cells /tubule
G
H
I
J
K
L
XII
I

Fig. S3. Generation of the *Katnal1*$^{GCKO/GCKO}$ mouse model

Katnal1 gene (A) and transcript structure (B). Red arrows show the approximate position of the *LoxP* insertion sites in the *Katnal1*$^{Flox/Flox}$ mice. The MT interacting and transport (MIT) domain, AAA ATPase domain and VPS4 domain are shown in green, blue and yellow respectively. Untranslated regions are shown in grey. (C) qPCR analysis of *Katnal1* transcript levels in *Katnal1*$^{GCKO/GCKO}$ isolated spermatocytes relative to *Katnal1*$^{Flox/Flox}$ controls (n=3/genotype). Data are normalised to *Ppia,* and lines represent mean ±s.d. ****P<0.0001. (D) Western blot analysis of KATNAL1 in isolated spermatocytes from *Katnal1*$^{Flox/Flox}$ and *Katnal1*$^{GCKO/GCKO}$ mice. Blots were re-probed with actin as a loading control. (E) Testis sections immunolabeled for KATNAL1 (green) in *Katnal1*$^{Flox/Flox}$ and *Katnal1*$^{GCKO/GCKO}$ mice. DNA and acrosomes were counterstained with DAPI (blue) and PNA (magenta) respectively. The average number of cleaved-caspase 3/7 positive germ cells per seminiferous tubule is graphed in (F) and representative images are shown in G-L. In F lines represent mean±s.d. A minimum of 80 tubules per mouse were counted (n≥3/genotype). Scale bars in E = 20 µm, G-H= 50 µm, I-L = 10 µm.

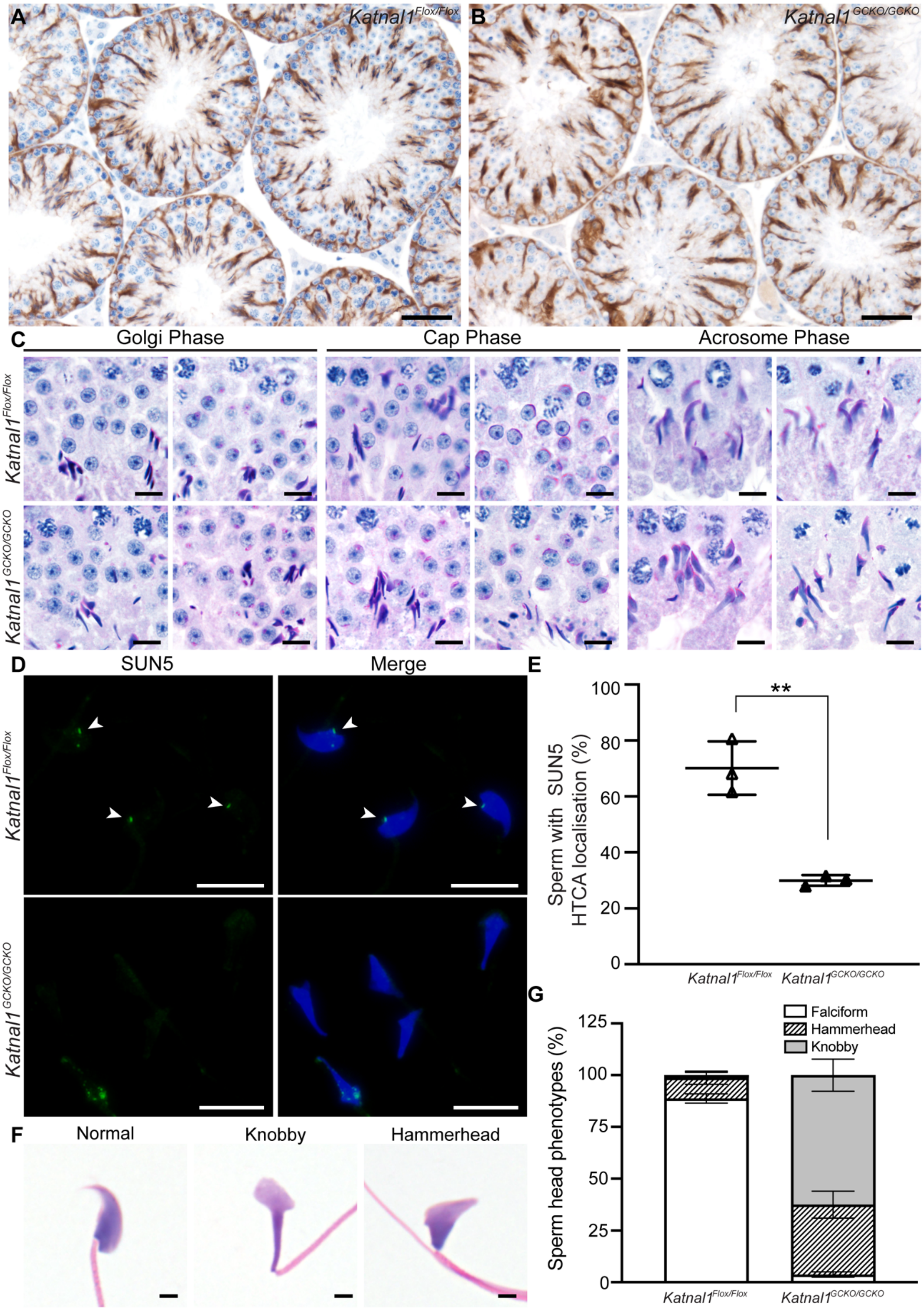
A
Katnal1 Flox/Flox
B
Katnal1 GCKO/GCKO
C
Golgi Phase
Cap Phase
Acrosome Phase
Katnal1 Flox/Flox
Katnal1 GCKO/GCKO
D
SUN5
Merge
Katnal1 Flox/Flox
Katnal1 GCKO/GCKO
E
Sperm with SUN5 HTCA localisation (%)
**
Katnal1 Flox/Flox
Katnal1 GCKO/GCKO
F
Normal
Knobby
Hammerhead
G
Sperm head phenotypes (%)
Falciform
Hammerhead
Knobby
Katnal1 Flox/Flox
Katnal1 GCKO/GCKO

Fig. S4. ***Katnal1$^{GCKO/GCKO}$*** **mice contain a normal Sertoli cell MT cytoskeleton and acrosome formation but have defects in sperm head shape and HTCA integrity**

Katnal1$^{Flox/Flox}$ (A) and *Katnal1$^{GCKO/GCKO}$* (B) testis sections immunolabelled for the Sertoli cell specific β-tubulin isoform TUBB3. (C) PAS-stained testis sections showing acrosome formation in *Katnal1$^{Flox/Flox}$* and *Katnal1$^{GCKO/GCKO}$* mice. Progressive steps of acrosome formation are shown left to right. Roman numerals denote seminiferous tubule stage. (D) Representative images of epididymal sperm from *Katnal1$^{Flox/Flox}$* and *Katnal1$^{GCKO/GCKO}$* mice immunolabelled for SUN5 (green) as an essential component of the HTCA. Nuclei are counterstained with DAPI (blue). White arrowheads indicate SUN5 staining at the HTCA. (E) Percentage of epididymal that had SUN5 localised to the base of sperm head sperm in *Katnal1$^{Flox/Flox}$* (white triangles) and *Katnal1$^{GCKO/GCKO}$* (black triangles) mice (n≥3/genotype, for each animal and a minimum of 100 epididymal sperm per animal, lines represent mean±s.d.). Example images of sperm head shape phenotypes (F) and percentage (±s.d.) of each sperm head phenotype in *Katnal1$^{Flox/Flox}$* and *Katnal1$^{GCKO/GCKO}$* mice (G) (n=4/genotype, for each animal a minimum of 100 epididymal sperm were assessed). $^{**}P$<0.01. Scale bars in A = 50 µm, B, D = 10 µm, F = 2 µm.

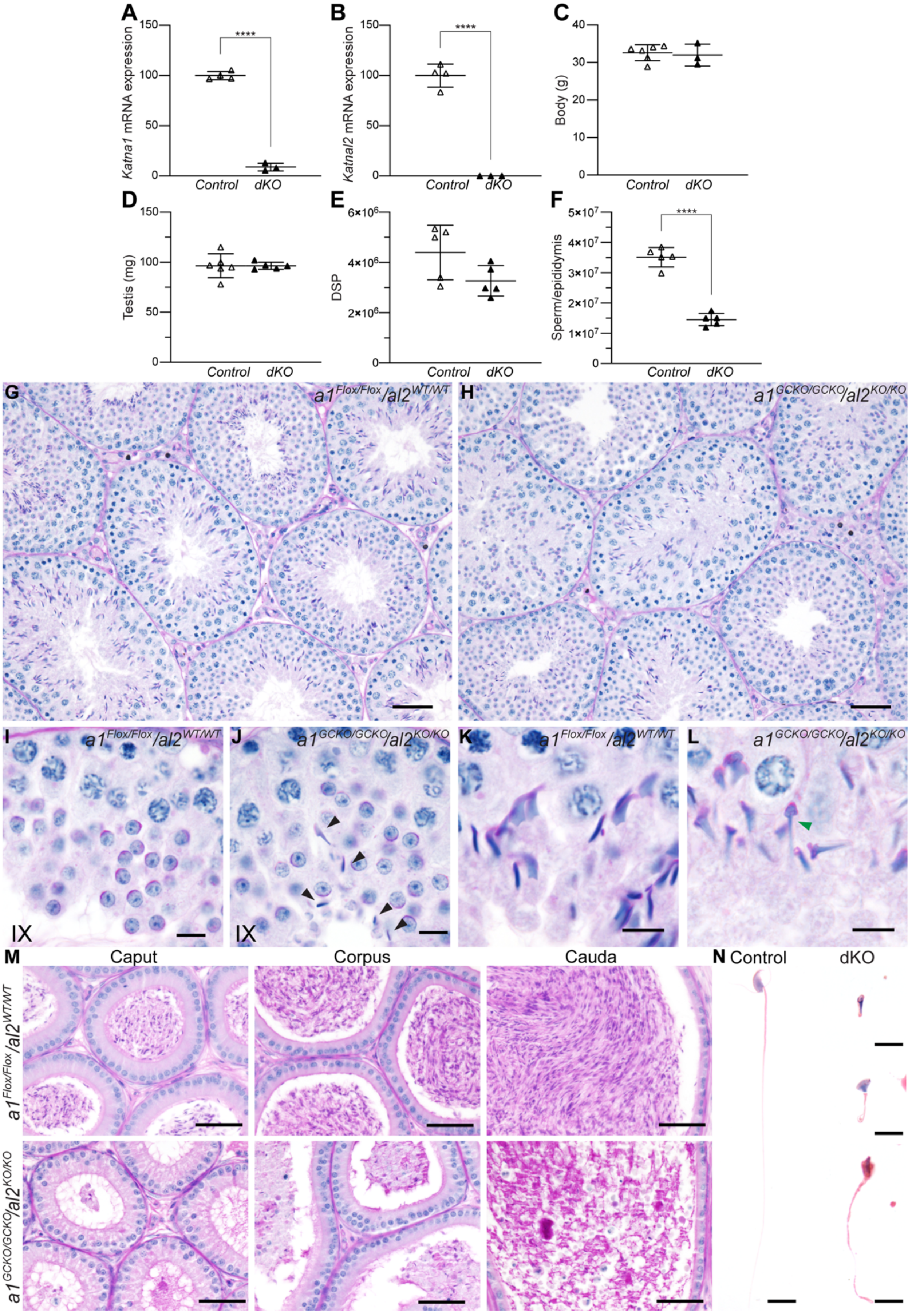
A
Katna1 mRNA expression
150
100
50
0
****
Control
dKO
B
Katnal2 mRNA expression
150
100
50
0
****
Control
dKO
C
Body (g)
40
30
20
10
0
Control
dKO
D
Testis (mg)
150
100
50
0
Control
dKO
E
DSP
6×10^6
4×10^6
2×10^6
0
Control
dKO
F
Sperm/epididymis
5×10^7
4×10^7
3×10^7
2×10^7
1×10^7
0
****
Control
dKO
G
a1 Flox/Flox /al2 WT/WT
H
a1 GCKO/GCKO /al2 KO/KO
I
a1 Flox/Flox /al2 WT/WT
IX
J
a1 GCKO/GCKO /al2 KO/KO
IX
K
a1 Flox/Flox /al2 WT/WT
L
a1 GCKO/GCKO /al2 KO/KO
M
Caput
Corpus
Cauda
a1 Flox/Flox /al2 WT/WT
a1 GCKO/GCKO /al2 KO/KO
N
Control
dKO

Fig. S5. KATNA1 and KATNAL2 do not function redundantly during mouse spermatogenesis

qPCR analysis of *Katna1* (A) and *Katnal2* (B) transcript levels in $Katna1^{GCKO/GCKO}/al2^{KO/KO}$ (black triangles) isolated spermatocytes relative to $Katna1^{Flox/Flox}/al2^{WT/WT}$ (white triangles) isolated spermatocytes (n≥3/genotype). Body weight (C), testis weight (D), testis DSP (E), and epididymal sperm content (F) in $Katna1^{GCKO/GCKO}/al2^{KO/KO}$ mice (black triangles) compared to $Katna1^{Flox/Flox}/al2^{WT/WT}$ controls (white triangles) (n≥3/genotype). Error bars represent mean±s.d. PAS-stained testis (G-L) and epididymis (M) sections, and hematoxylin and eosin-stained cauda epididymal sperm (N), from $Katna1^{GCKO/GCKO}/al2^{KO/KO}$ and $Katna1^{Flox/Flox}/al2^{WT/WT}$ mice. Scale bars = 50 µm in G-H, 10 µm in I-L, 40 µm in M-N. ****P<0.0001.

Development • Supplementary information

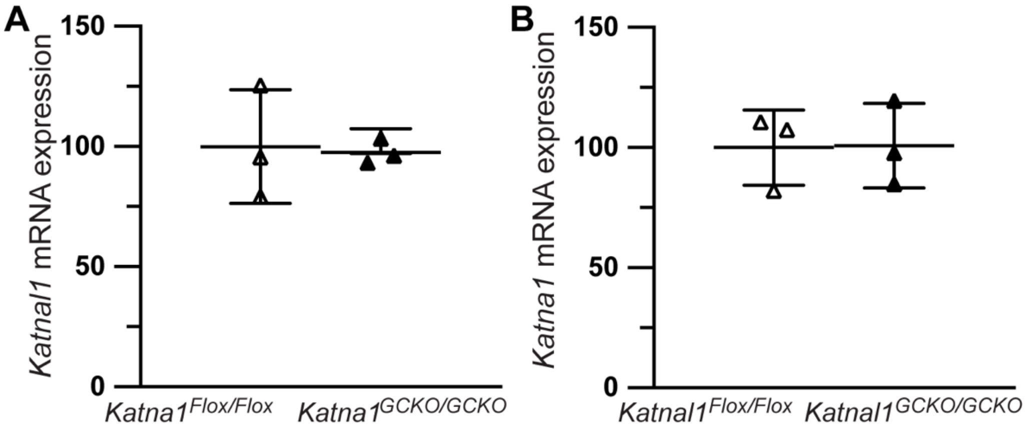

Fig. S6. Functional compensation between KATNA1 and KATNAL1 is not mediated by gene upregulation.

(A) qPCR analysis of *Katnal1* transcript levels in *Katna1*$^{GCKO/GCKO}$ (black triangles) relative to *Katna1*$^{Flox/Flox}$ (white triangles) isolated spermatocytes (n=3/genotype). (B) qPCR analysis of *Katna1* transcript levels in *Katnal1*$^{GCKO/GCKO}$ (black triangles) relative to *Katnal1*$^{Flox/Flox}$ (white triangles) isolated spermatocytes (n=3/genotype). Data are normalised to *Ppia,* and lines represent mean±s.d.

Development • Supplementary information

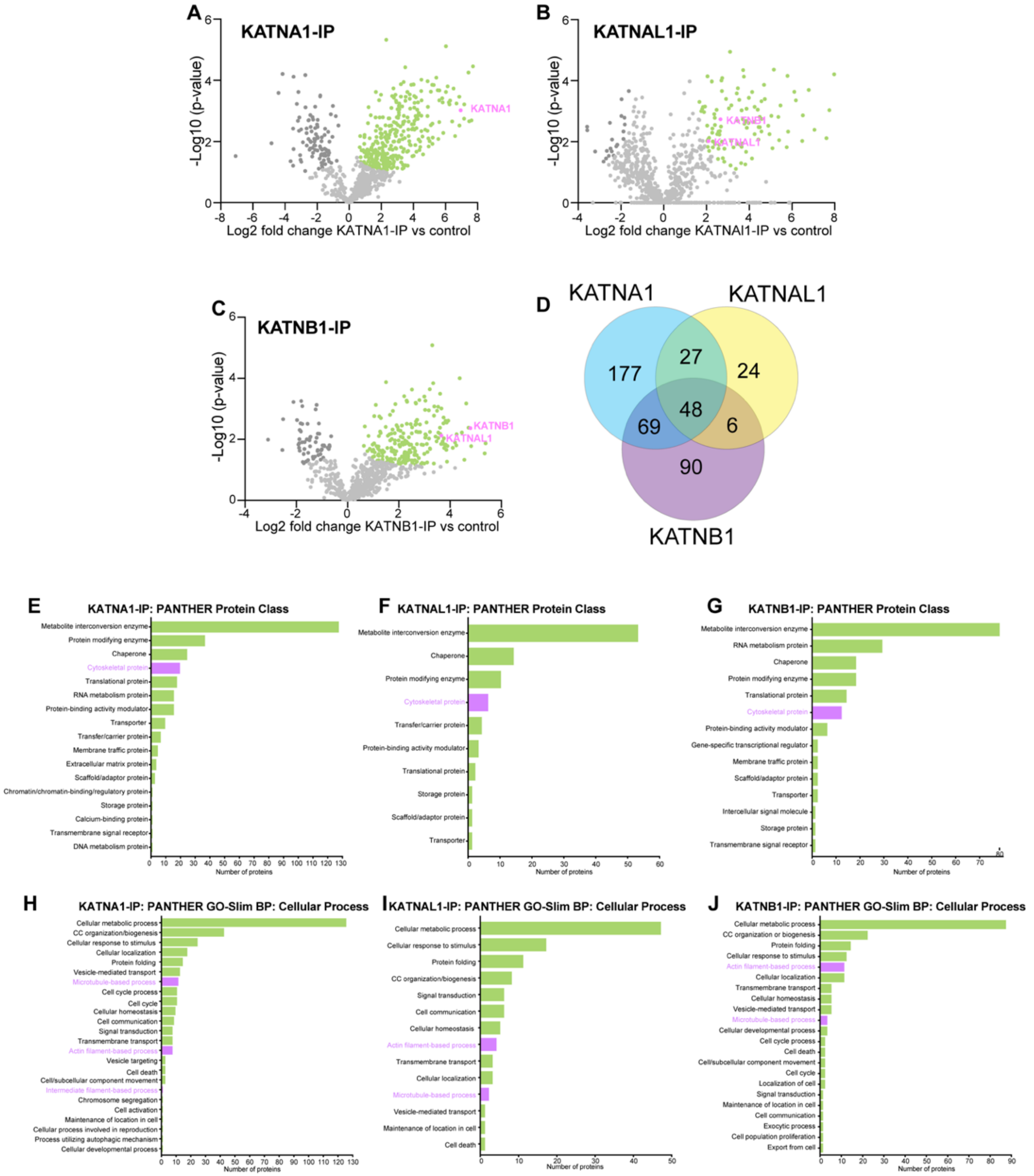

A
KATNA1-IP
-Log10 (p-value)
KATNA1
Log2 fold change KATNA1-IP vs control
B
KATNAL1-IP
-Log10 (p-value)
KATNB1
KATNAL1
Log2 fold change KATNAl1-IP vs control
C
KATNB1-IP
-Log10 (p-value)
KATNB1
KATNAL1
Log2 fold change KATNB1-IP vs control
D
KATNA1
KATNAL1
177
27
24
48
69
6
90
KATNB1
E
KATNA1-IP: PANTHER Protein Class
Metabolite interconversion enzyme
Protein modifying enzyme
Chaperone
Cytoskeletal protein
Translational protein
RNA metabolism protein
Protein-binding activity modulator
Transporter
Transfer/carrier protein
Membrane traffic protein
Extracellular matrix protein
Scaffold/adaptor protein
Chromatin/chromatin-binding/regulatory protein
Storage protein
Calcium-binding protein
Transmembrane signal receptor
DNA metabolism protein
Number of proteins
F
KATNAL1-IP: PANTHER Protein Class
Metabolite interconversion enzyme
Chaperone
Protein modifying enzyme
Cytoskeletal protein
Transfer/carrier protein
Protein-binding activity modulator
Translational protein
Storage protein
Scaffold/adaptor protein
Transporter
Number of proteins
G
KATNB1-IP: PANTHER Protein Class
Metabolite interconversion enzyme
RNA metabolism protein
Chaperone
Protein modifying enzyme
Translational protein
Cytoskeletal protein
Protein-binding activity modulator
Gene-specific transcriptional regulator
Membrane traffic protein
Scaffold/adaptor protein
Transporter
Intercellular signal molecule
Storage protein
Transmembrane signal receptor
Number of proteins
H
KATNA1-IP: PANTHER GO-Slim BP: Cellular Process
Cellular metabolic process
CC organization/biogenesis
Cellular response to stimulus
Cellular localization
Protein folding
Vesicle-mediated transport
Microtubule-based process
Cell cycle process
Cell cycle
Cellular homeostasis
Cell communication
Signal transduction
Transmembrane transport
Actin filament-based process
Vesicle targeting
Cell death
Cell/subcellular component movement
Intermediate filament-based process
Chromosome segregation
Cell activation
Maintenance of location in cell
Cellular process involved in reproduction
Process utilizing autophagic mechanism
Cellular developmental process
Number of proteins
I
KATNAL1-IP: PANTHER GO-Slim BP: Cellular Process
Cellular metabolic process
Cellular response to stimulus
Protein folding
CC organization/biogenesis
Signal transduction
Cell communication
Cellular homeostasis
Actin filament-based process
Transmembrane transport
Cellular localization
Microtubule-based process
Vesicle-mediated transport
Maintenance of location in cell
Cell death
Number of proteins
J
KATNB1-IP: PANTHER GO-Slim BP: Cellular Process
Cellular metabolic process
CC organization or biogenesis
Protein folding
Cellular response to stimulus
Actin filament-based process
Cellular localization
Transmembrane transport
Cellular homeostasis
Vesicle-mediated transport
Microtubule-based process
Cellular developmental process
Cell cycle process
Cell death
Cell/subcellular component movement
Cell cycle
Localization of cell
Signal transduction
Maintenance of location in cell
Cell communication
Exocytic process
Cell population proliferation
Export from cell
Number of proteins

Fig. S7. Identification of KATNA1, KATNAL1 and KATNAL2 candidate testis interaction partners

Volcano blots showing the statistical enrichment of protein groups identified in the KATNA1 (A), KATNAL1 (B) and KATNB1 (C) IP-MS assays. All protein groups identified, IP-MS measurements and statistical analysis is provided in Dataset S1. Protein groups shown in green or pink, represent those significantly enriched in the experimental IP (KATNA1, KATNAL1 or KATNB1). For each IP-MS assay n=3 biological replicates were assessed. Venn diagram (D) showing the overlap in the identity of the protein groups identified in each of the KATNA1, KATNAL1 and KATNB1 IP-MS assays. PANTHER Protein Class and Gene Ontology analysis was used to analyse the proteins significantly enriched in each of the KATNA1, KATNAL1 and KATNB1 IPs (E-J). The PANTHER Protein Class (E-F) and the of the PANTHER GO-Slim Biological Process (BP) Cellular Process (GO:0009987) sub-classification (H-J) assigned to the proteins significantly enriched in each of the KATNA1, KATNAL1 and KATNB1 IPs. PANTHER was not able to assign all proteins identified. Detailed PANTHER Protein Class and GO-Slim analysis is provided in Dataset S2.

Table S1. Significantly enriched proteins identified in KATNA1, KATNAL1 and KATNB1 testis co-immunoprecipitation experiments with subsequent mass spectrometry analysis.

Available for download at
https://journals.biologists.com/dev/article-lookup/doi/10.1242/dev.201956#supplementary-data

Table S2. PANTHER analysis of proteins identified by mass spectrometry as significantly enriched in either KATNA1, KATNAL1 or KATNB1 testis co-immunoprecipitates.

Available for download at
https://journals.biologists.com/dev/article-lookup/doi/10.1242/dev.201956#supplementary-data

Table S3. Functional descriptions of selected KATNA1 (A1), KATNAL1 (AL1) and KATNB1 (B1) candidate testis binding proteins of interest.

Available for download at
https://journals.biologists.com/dev/article-lookup/doi/10.1242/dev.201956#supplementary-data

Development • Supplementary information

Supplemental references

Adams, M., Simms, R. J., Abdelhamed, Z., Dawe, H. R., Szymanska, K., Logan, C. V., Wheway, G., Pitt, E., Gull, K., Knowles, M. A., et al. (2012). A meckelin-filamin A interaction mediates ciliogenesis. *Hum Mol Genet* **21**, 1272-1286.

Barbosa, D. J., Teixeira, V., Duro, J., Carvalho, A. X. and Gassmann, R. (2021). Dynein-dynactin segregate meiotic chromosomes in C. elegans spermatocytes. *Development* **148**.

Chan, P. C., Hsu, R. Y., Liu, C. W., Lai, C. C. and Chen, H. C. (2014). Adducin-1 is essential for mitotic spindle assembly through its interaction with myosin-X. *J Cell Biol* **204**, 19-28.

Da Costa, R., Bordessoules, M., Guilleman, M., Carmignac, V., Lhussiez, V., Courot, H., Bataille, A., Chlémaire, A., Bruno, C., Fauque, P., et al. (2020). Vps13b is required for acrosome biogenesis through functions in Golgi dynamic and membrane trafficking. *Cellular and Molecular Life Sciences* **77**, 511-529.

Dix, D. J., Allen, J. W., Collins, B. W., Mori, C., Nakamura, N., Poorman-Allen, P., Goulding, E. H. and Eddy, E. M. (1996). Targeted gene disruption of Hsp70-2 results in failed meiosis, germ cell apoptosis, and male infertility. *Proceedings of the National Academy of Sciences* **93**, 3264-3268.

Dix, D. J., Allen, J. W., Collins, B. W., Poorman-Allen, P., Mori, C., Blizard, D. R., Brown, P. R., Goulding, E. H., Strong, B. D. and Eddy, E. M. (1997). HSP70-2 is required for desynapsis of synaptonemal complexes during meiotic prophase in juvenile and adult mouse spermatocytes. *Development* **124**, 4595-4603.

Fouquet, J., Kann, M., Soues, S. and Melki, R. (2000). ARP1 in Golgi organisation and attachment of manchette microtubules to the nucleus during mammalian spermatogenesis. *Journal of Cell Science* **113**, 877-886.

Govin, J., Caron, C., Escoffier, E., Ferro, M., Kuhn, L., Rousseaux, S., Eddy, E. M., Garin, J. and Khochbin, S. (2006). Post-meiotic shifts in HSPA2/HSP70.2 chaperone activity during mouse spermatogenesis. *J Biol Chem* **281**, 37888-37892.

Hsu, W. H., Wang, W. J., Lin, W. Y., Huang, Y. M., Lai, C. C., Liao, J. C. and Chen, H. C. (2018). Adducin-1 is essential for spindle pole integrity through its interaction with TPX2. *EMBO Rep* **19**.

Kanemori, Y., Koga, Y., Sudo, M., Kang, W., Kashiwabara, S.-i., Ikawa, M., Hasuwa, H., Nagashima, K., Ishikawa, Y. and Ogonuki, N. (2016). Biogenesis of sperm acrosome is regulated by pre-mRNA alternative splicing of Acrbp in the mouse. *Proceedings of the National Academy of Sciences* **113**, E3696-E3705.

Lee, N. P. Y., Mruk, D. D., Conway, A. M. and Cheng, C. Y. (2004). Zyxin, Axin, and Wiskott-Aldrich Syndrome Protein Are Adaptors That Link the Cadherin/Catenin Protein Complex to the Cytoskeleton at Adherens Junctions in the Seminiferous Epithelium of the Rat Testis. *Journal of Andrology* **25**, 200-215.

Li, N., Mruk, D. D., Tang, E. I., Wong, C. K. C., Lee, W. M., Silvestrini, B. and Cheng, C. Y. (2015). Formins: Actin nucleators that regulate cytoskeletal dynamics during spermatogenesis. *Spermatogenesis* **5**, e1066476.

Lie, P. P., Chan, A. Y., Mruk, D. D., Lee, W. M. and Cheng, C. Y. (2010). Restricted Arp3 expression in the testis prevents blood–testis barrier disruption during junction restructuring at spermatogenesis. *Proceedings of the National Academy of Sciences* **107**, 11411-11416.

Lin, Y.-H., Huang, C.-Y., Ke, C.-C., Wang, Y.-Y., Lai, T.-H., Liu, H.-C., Ku, W.-C., Chan, C.-C. and Lin, Y.-H. (2020). ACTN4 mediates SEPT14 mutation-induced sperm head defects. *Biomedicines* **8**, 518.

Liu, L., Zhang, Y., Chang, X., Li, R., Wu, C., Tang, L. and Zhou, Z. (2018). Fluorochloridone perturbs blood-testis barrier/Sertoli cell barrier function through Arp3-mediated F-actin disruption. *Toxicology Letters* **295**, 277-287.

Morohoshi, A., Miyata, H., Oyama, Y., Oura, S., Noda, T. and Ikawa, M. (2021). FAM71F1 binds to RAB2A and RAB2B and is essential for acrosome formation and male fertility in mice. *Development* **148**.

Mountjoy, J. R., Xu, W., McLeod, D., Hyndman, D. and Oko, R. (2008). RAB2A: a major subacrosomal protein of bovine spermatozoa implicated in acrosomal biogenesis. *Biol Reprod* **79**, 223-232.

O'Donnell, L., Nicholls, P. K., O'Bryan, M. K., McLachlan, R. I. and Stanton, P. G. (2011). Spermiation: the process of sperm release. *Spermatogenesis* **1**, 14-35.

Qi, Y., Jiang, M., Yuan, Y., Bi, Y., Zheng, B., Guo, X., Huang, X., Zhou, Z. and Sha, J. (2013). ADP-ribosylation factor-like 3, a manchette-associated protein, is essential for mouse spermiogenesis. *Molecular Human Reproduction* **19**, 327-335.

Ramalho-Santos, J., Moreno, R. D., Wessel, G. M., Chan, E. K. L. and Schatten, G. (2001). Membrane Trafficking Machinery Components Associated with the Mammalian Acrosome during Spermiogenesis. *Experimental Cell Research* **267**, 45-60.

Sangokoya, C. and Blelloch, R. (2020). MicroRNA-dependent inhibition of PFN2 orchestrates ERK activation and pluripotent state transitions by regulating endocytosis. *Proc Natl Acad Sci U S A* **117**, 20625-20635.

Schöpp, T., Zoch, A., Berrens, R. V., Auchynnikava, T., Kabayama, Y., Vasiliauskaitė, L., Rappsilber, J., Allshire, R. C. and O'Carroll, D. (2020). TEX15 is an essential executor of MIWI2-directed transposon DNA methylation and silencing. *Nature Communications* **11**, 3739.

Selvaraj, V., Asano, A., Page, J. L., Nelson, J. L., Kothapalli, K. S. D., Foster, J. A., Brenna, J. T., Weiss, R. S. and Travis, A. J. (2010). Mice lacking FABP9/PERF15 develop sperm head abnormalities but are fertile. *Developmental Biology* **348**, 177-189.

Tang, E. I., Mok, K. W., Lee, W. M. and Cheng, C. Y. (2015). EB1 regulates tubulin and actin cytoskeletal networks at the sertoli cell blood-testis barrier in male rats: an in vitro study. *Endocrinology* **156**, 680-693.

Tarkar, A., Loges, N. T., Slagle, C. E., Francis, R., Dougherty, G. W., Tamayo, J. V., Shook, B., Cantino, M., Schwartz, D., Jahnke, C., et al. (2013). DYX1C1 is required for axonemal dynein assembly and ciliary motility. *Nature Genetics* **45**, 995-1003.

Wang, F., Zhang, Q., Cao, J., Huang, Q. and Zhu, X. (2008). The microtubule plus end-binding protein EB1 is involved in Sertoli cell plasticity in testicular seminiferous tubules. *Experimental Cell Research* **314**, 213-226.

Wu, C.-H., Zong, Q., Du, A.-L., Zhang, W., Yao, H.-C., Yu, X.-Q. and Wang, Y.-F. (2016). Knockdown of Dynamitin in testes significantly decreased male fertility in Drosophila melanogaster. *Developmental biology* **420**, 79-89.

Yang , F., Eckardt , S., Leu , N. A., McLaughlin , K. J. and Wang , P. J. (2008). Mouse TEX15 is essential for DNA double-strand break repair and chromosomal synapsis during male meiosis. *Journal of Cell Biology* **180**, 673-679.

Yuan, S., Stratton, C. J., Bao, J., Zheng, H., Bhetwal, B. P., Yanagimachi, R. and Yan, W. (2015). Spata6 is required for normal assembly of the sperm connecting piece and tight head-tail conjunction. *Proc Natl Acad Sci U S A* **112**, E430-439.
