# Supplementary material for: The katanin A-subunits KATNA1 and KATNAL1 act co-operatively in mammalian meiosis and spermiogenesis to achieve male fertility
Source: Development. 2023 Nov 13;150(22):dev201956. doi: 10.1242/dev.201956 (PMC10690054; doi:10.1242/dev.201956)
Supplement: Supplementary file 1 [file develop-150-201956_review_history.pdf]

The katanin A-subunits KATNA1 and KATNAL1 act co-operatively in mammalian meiosis and spermiogenesis to achieve male fertility

Jessica E.M. Dunleavy, Maddison Graffeo, Kathryn Wozniak, Anne E. O'Connor, D. Jo Merriner, Joseph Nguyen, Ralf B. Schittenhelm, Brendan J. Houston and Moira K. O'Bryan

DOI: 10.1242/dev.201956

Editor: Haruhiko Koseki

Review timeline

Original submission:	7 May 2023
Editorial decision:	7 June 2023
First revision received:	21 September 2023
Editorial decision:	3 October 2023
Second revision received:	3 October 2023
Accepted:	10 October 2023

Original submission

First decision letter

MS ID#: DEVELOP/2023/201956

MS TITLE: Katanin A-subunits KATNA1 and KATNAL1 act co-operatively in mammalian meiosis and spermiogenesis to achieve male fertility

AUTHORS: Jessica EM Dunleavy, Maddison Graffeo, Kathryn Wozniak, Anne O'Connor, D. Jo Merriner, Joseph Nguyen, Ralf B Schittenhelm, Brendan Houston, and Moira K O'Bryan

I have now received all the referees' reports on the above manuscript, and have reached a decision. The referees' comments are appended below, or you can access them online: please go to BenchPress and click on the 'Manuscripts with Decisions' queue in the Author Area.

As you will see, the referees express considerable interest in your work, but have some significant criticisms and recommend a substantial revision of your manuscript before we can consider publication. If you are able to revise the manuscript along the lines suggested, which may involve further experiments, I will be happy receive a revised version of the manuscript. Your revised paper will be re-reviewed by one or more of the original referees, and acceptance of your manuscript will depend on your addressing satisfactorily the reviewers' major concerns. Please also note that Development will normally permit only one round of major revision. If it would be helpful, you are welcome to contact us to discuss your revision in greater detail. Please send us a point-by-point response indicating your plans for addressing the referees' comments, and we will look over this and provide further guidance.

Please attend to all of the reviewers' comments and ensure that you clearly highlight all changes made in the revised manuscript. Please avoid using 'Tracked changes' in Word files as these are lost in PDF conversion. I should be grateful if you would also provide a point-by-point response detailing how you have dealt with the points raised by the reviewers in the 'Response to Reviewers' box. If you do not agree with any of their criticisms or suggestions please explain clearly why this is so.

Reviewer 1

© 2023. Published by The Company of Biologists under the terms of the Creative Commons Attribution License (https://creativecommons.org/licenses/by/4.0/).

Advance summary and potential significance to field

Meiosis and spermiogenesis are consecutive developmental processes that enable spermatogenic cells to acquire proper genome and morphology to become functional spermatozoa. Abnormalities that occur during these processes are often detrimental to sperm production and are direct causes underlying human reproductive and genetic diseases. Intracellular cytoskeletal systems undergo coordinated dynamic changes during meiosis and spermiogenesis, however, how it is regulated remain largely unclear. The authors of this manuscript elucidated the functional roles of a family of important microtubule severing enzyme, Katanin using mouse genetics, as well as cell biological and biochemical experiments. Their data showed that the catalytic subunits KATNA1 and KATNAL1 of Katanin complex (composed of catalytic A and regulatory B subunits) play essential roles during meiosis and spermiogenesis. Specifically, deletion of KATNAL1 or both KATNA1 and KATNAL1 disrupted numerous aspects of meiotic division and post-meiotic development, most of which are likely due to the compromised regulation of microtubule dynamics, including the formation of meiotic spindle, acrosome, nucleus, mitochondria sheath and flagella of sperm tail. These data add complementary information to previous studies of Katanin family during spermatogenesis and provide new foundations to understand the complex and coordinated cellular processes that spermatogenic cells undertake during their development.

Comments for the author

For most part of the manuscript, the phenotypes of mutant mice are clearly presented. The histological and electron microscopic images are of good quality. However, the mechanistic insights obtained from the data tend to be speculative. Some aspects of the manuscript are less clear and additional experiments may be needed for the conclusions drawn to be more convincing. These include:

1. It'll be helpful for the understanding of compensatory effects of Katanin subunits if their expression patterns during spermatogenesis could be introduced and compared, including that of KATNA1, KATNAL1 KATNAL2 and KATNB1. Since no obvious phenotype was observed for Katna1-CKO and dual deletion of Katna1 and Katnal1 showed more aggressive phenotypes than the deletion of Katnal1, what are the expression changes of the two genes in respective single mutants? If Katnal1-CKO showed severe phenotypes during meiosis and spermiogenesis, it may not suggest that "KATNA1 and KATNAL1 compensate for each other" (line 291) or "KATNA1 is dispensable for mammalian spermatogenesis" (line 117).

2. Were the axonemal structures examined for Katna1/Katnal1-DCKO mice using transmission electron microscopy?

3. Can the Co-IP/MS results be verified? For example, although the links to Actin cytoskeleton is interesting would immunostaining of Actin reveal differences between wild type and KO sperm? Some Western blotting following Co-IP for proteins in the mentioned interactomes would be helpful.

4. The Stra8-Cre mouse lines used should be mentioned in the main text when introducing Katna1-GCKO and Katnal1-GCKO. Can Western blotting used to show the protein products in Katna1-GCKO mice if any (lines 106-107)?

5. Figure S3A, what the red arrow heads show? Indicate positions for sgRNAs, LoxPs, PCR primers for genotyping and scheme of resulting alleles; indicate deletion of ATPase and VPS4 domains? Off-target testing? Figure S3E, how this staining is compared to that of Smith, et al., 2012 showing Sertoli specific expression? Is this due to the different antibodies used?

6. The Materials and Methods section need to be more thorough and clearer, especially on, how the antibodies are used (Antibody use, Western blotting, Immunochemistry and Imaging sections should be re-organized), how DSP is calculated, how the morphological defects in Katnal1-CKO are measured, how significant proteins are determined following MS. The statistics should be presented with s.d. and p values in the main text (lines 158-167, lines 315-316).

© 2023. Published by The Company of Biologists under the terms of the Creative Commons Attribution License (https://creativecommons.org/licenses/by/4.0/).

Some minor concerns:

1. Instead of using “GCKO” in most of the figures, specific genes affected should be shown, for example Katnal1-GCKO, in order to improve the readability.

2. Lines 146-148 described Sertoli-only tubules, which is hardly shown in Fig.1F.

3. Indicate midbodies in Fig. 2Ac, g and Fig. 5Ag, k (lines 348-349) with arrows;

4. Line 1150, blue or green arrowheads?

5. Line 420, to show the significance determined by what criteria?

Reviewer 2

Advance summary and potential significance to field

The paper by Jessica Dunleavy et al. entitled “Katanin A-subunits KATNA1 and KATNAL1 act cooperatively in mammalian meiosisand spermiogenesis to achieve male fertility” describes the establishment and analysis of Katna1 and Kantal1 deficient mouse lines. The authors report many pathophysiological changes and perform a wide set matings in order to decipher common and compensatory effects of microtubule severing proteins.

Comments for the author

While Dunelavy et al. report interesting phenotypes, which adds to the understanding of the genes required for spermatogenesis the authors need to address some points before the ms should be considered for publication. Further, the reviewer would like to mention, that reporting on aberrations of sperm in epididymis from such animals as primary effect of the mutation is tricky. Obviously, spermatogenesis and spermiogenesis are affected at earlier stages leading to initiation of cell death – hence, what the authors are observing might represent a secondary effect. This option should be mentioned and discussed.

In detail:

1.) line 133 – Fig 1D does not show apoptotic germ cells as stated in the text. Pls. revise.
2.) line 158 ff. paragraph describing morphologically abnormal sperm in Katnl1 gko mice. To the reviewer’s opinion, this defect could very well be a secondary effect, since many murine models showing problems in spermiogenesis show malformed sperm owing to sperm degradation in the epididymis. Either, the authors water the claim down and include the possibility of this being a secondary effect, or they present pictures of malformed sperm in the testes.
3.) Figures are sometimes not listed in proper succession, e.g. Line 142 -Fig 1G and line 146 Fig E,F – There are more of such examples – this makes reading difficult. Pls. re-organize the figures accordingly.
4.) line 164 – “the ability for motility” sounds odd – why not “reduction in motility”
5.) line 169 – the claim is too general, it needs to be more specific.
6.) Fig 2A – would benefit from a fluorescent staining method, alternatively, please add some (bigger) arrowheads to pinpoint what you describe in order for the non-meiosis aficionado get your point.
7.) Fig. 2D seems not mentioned in the text.
8.) Fig. 2 E-J are only mentioned in one half sentence together with Fig 2A (line 184). Either explain to the reader what is there to see or move them to supplemental.
9.) Line 193 and 198 the reviewer reads some relative terms such as “often observed” or “a subset of the resulting spermatids”. Could the authors try to count these observations and add them to the (then amended) text?
10.) Fig. 3A and B – is a blurry and too little enlarged photograph. The reviewer suggests to take pictures at higher magnification and probably add the overview picture as supplemental material.

© 2023. Published by The Company of Biologists under the terms of the Creative Commons Attribution License (https://creativecommons.org/licenses/by/4.0/).

11.) Line 226 ff. Fig. 3C. Again, the reviewer stresses the point that this could be a secondary effect of the degradation process. This possibility must be mentioned, when presenting the data. Alternatively, sections from testes demonstrating such defects would suffice to make the point.
12.) Line 235. The authors use here the term “wild-type” – to be correct it is material from flox/flox – mice. They might be considered “controls” but the term wild-type might be misleading.
13.) Line 246 ff. refers to point 11 – consider revising owing to this potentially being a secondary defect.
14.) Line 261 – The authors state “In some cells..” – please quantify “some” to substantiate the claim and give the reader an impression of the extent of the sperm heads altered.
15.) Line 265 – SUN5 Immunofluorescence must be repeated in order to give a result, which one can interpret. At present the picture is too dark/staining too weak to be able to interpret.
16.) Line 270-271 – this claim seems not backed by the data – could be presented as speculation.
17.) Line 282 “dissembled” – should it read “disassembled”?
18.) Line 287 Fig 3F – could the pictures of control and the mutant be taken with the same intensity/white balance in order to be able to compare the staining. Also, manchette development is really much better presented by using fluorescent labelling (PNA-FITC or comparable). The reviewer thinks that replacing the present pictures by fluorescent ones would improve the ability to judge the claims made by the authors.
19.) Line 291 ff – paragraph discusses the compensation of both proteins deleted. However, in technical terms, this claim is made by assessing anatomy and morphology. There are no molecular data such as qRT-PCR or Western Blot in order to look for levels of transcripts or proteins in the respective ko mice. Maybe there is a compensation i.e. upregulation, but this is not sufficient. The authors should consider mention this in the text or provide experiments to substantiate the claim.
20.) line 304 – the authors should make it clear that with this sentence “No aspect..” they refer not only to their results presented here, but also to the previous publication they mention before.
21.) line 310 – “Successful” ablation of…Please delete “successful” since it is meaningless.
22.) line 321 – Fig. 4g-H is too small magnification to really see all the details. A higher magnification must be provided. Further, the arrowheads are too small and the colors of the arrows is unfortunate so that they are only hard to see. Please revise this part of the figure.
23.) line 355-357 – the authors claim that the frequency of binucleated spermatids…. was similar to….The reviewer wonders where to find the data (numbers) and the respective statistics for this claim. Please add.
24.) line 359: typo spermiogenesisrevealed
25.) line 360 – the analysis of acrosome biogenesis (Fig 6 A,B) would benefit from a immunofluorescent staining. Alternatively, higher mag insets could make the point as well. Blue arrowhead is hard to see – consider other color.
26.) line 389 Fig 6G manchette analysis would benefit from immunofluorescent labelling
27.) line 414 ff – the authors finally perform an IP and a MS analysis to detect potential interaction partners.
To the reviewer this paragraph, while interesting has some shortcomings. First, none of the detected proteins are validated by a further experiment (CoIP, reverse IP), second the candidate mentioned before SUN5 (see point 7) seems not detected or is at least not mentioned in the main text. Second – the MS analysis detects protein (fragments) which are pulled down in such an experiment. However, without further biochemical analyses, one cannot state that protein x “is bound to” KATNA1 etc. Since there is the potential, that the pull-down reflects a larger protein complex “bound” can not be claimed here. Further, although, the authors filtered the data, there is always the potential of a backgound noise, which one only gets rid off by performing further experiments to validate the interaction. So, in sum, the last part appears rather weak and should be strengthened by validation experiments. Alternatively, since it might extend beyond the scope of this manuscript, the MS data could be considered to be left out to serve as a starting point for a (more biochemical) follow up study.

Reviewer 3

Advance summary and potential significance to field

The authors have conducted a study on the function of Katanin A-subunit protein in male germ cells using conditional KO mice. The data presented in the study have been diligently analyzed and the results are quite convincing. However, the manuscript could benefit from some trimming as it is

© 2023. Published by The Company of Biologists under the terms of the Creative Commons Attribution License (https://creativecommons.org/licenses/by/4.0/).

quite lengthy and some parts tend to blur the main focus. If the authors remove unnecessary parts of the sentences, the paper would become more readable and effective.

Comments for the author

Major points
On line 221, the authors mention that Katnal1 KO has abnormal sperm tails, but no quantitative data is provided. It would be helpful to have a clear illustration of the abnormal ratio of sperm tail in Katnal1 KO in Fig. 1J. Additionally, the KO's sperm midpiece defects seem to be around 10%, including coiled midpiece and mitochondrial sheath defects. This ratio is not significantly high. While the authors noted sperm tail abnormalities in Fig. 3C these data were obtained from the cauda epididymis. Therefore, to conclude that KATNAL1 is essential for "axoneme doublet formation" and "ODF/Mitochondrial sheath formation," the author must observe those organelles during spermiogenesis (testis). Since sperm obtained some stress during epididymal migration, and sperm in Fig. 3C may reflect secondary effects. To disprove this point, the author must place TEM pictures for step 16 spermatids in Fig. 3C. My guess is that abnormalities in step 16 spermatid tail are scarce. In that case it would be best not to make many mentions of the sperm tail since it is far from the main story of the paper.

Calculate the proportion of "misaligned/lagging chromosomes" in Fig. 5B, "binucleated round spermatids" in Fig. 5C, and abnormal acrosome vesicles in Fig. 6A? These findings are critical to this research paper.

Regarding sentences 440-486, they mostly constitute the discussion section. If you intend to incorporate them into the results section, it would be necessary to conduct further experiments. Thus, I suggest that these sentences be excluded from the paper.

Minor points
In the Results section, there is no reference to the use of Stra8-Cre Tg mice. These mice were utilized in the creation of all conditional KO mice mentioned in the study. It would be helpful to include this information in the Results section to aid the reader's comprehension.

The genes Katna1 and Katnal1 have such similar names that it can be difficult to tell them apart. While I understand that these are official gene symbols, could simpler names be used in the paper? The current names have caused confusion for me and other readers, adding unnecessary stress.

In line 138, based on the gathered data, would it be more appropriate to use "spermatogenesis" instead of "spermiogenesis" in the sentence "This reveals that gem cell derived KATNAL1 is required for optimal spermiogenesis and~"?

Line 155-156, “These data reveal that KATNAL1 plays an essential role in MT regulation in both Sertoli and germ cells” I think KATNAL1 is not essential for Sertoli cells from your data.

Line 205-209, I think this paragraph can be removed.

Line 303-306, Please include actual data from mating tests of Katna1/al2 double KO mouse line.

Line 351, Is Fig. 5Ag,k is correct? I think it should be Fig.5h,i.

Line 351-353, I think this sentence can be combined with the previous sentence.

Line 491, 492, “for the first time”, “first”. I think these expressions are not suitable for a research paper.

Fig. 1C, 4E, S2C, S5E, DSP is only spelled out in Materials and Methods. Please spell out in Line 134.

Fig. 3Cc, Fig. 3Ca-c should be pictures for midpiece, but Fig. 3Cc is a picture for principal piece.

© 2023. Published by The Company of Biologists under the terms of the Creative Commons Attribution License (https://creativecommons.org/licenses/by/4.0/).

Fig. 3F, In the process of spermiogenesis, does the Manchette of Katnal1 KO disappear? If it disappears, please put pictures until it disappears.

Fig.4, There are no pictures for sperm morphology of Katna1/al1 KO. This data is thought to be important.

First revision

Author response to reviewers' comments

Response to reviewers
We thank the reviewers for the time and effort they have put into reviewing our manuscript. We appreciate their insights. We have addressed each comment below and where possible have added new data and additional analyses of existing data. We feel these revisions we have addressed the requests and significantly improved the manuscript. We trust that is now acceptable for publication in Development.

Please note all line numbers refer to the version of the revised manuscript wherein changes are tracked.

Reviewer 1 Advance Summary and Potential Significance to Field:

Meiosis and spermiogenesis are consecutive developmental processes that enable spermatogenic cells to acquire proper genome and morphology to become functional spermatozoa. Abnormalities that occur during these processes are often detrimental to sperm production and are direct causes underlying human reproductive and genetic diseases. Intracellular cytoskeletal systems undergo coordinated dynamic changes during meiosis and spermiogenesis, however, how it is regulated remain largely unclear. The authors of this manuscript elucidated the functional roles of a family of important microtubule severing enzyme, Katanin, using mouse genetics, as well as cell biological and biochemical experiments. Their data showed that the catalytic subunits KATNA1 and KATNAL1 of Katanin complex (composed of catalytic A and regulatory B subunits) play essential roles during meiosis and spermiogenesis. Specifically, deletion of KATNAL1 or both KATNA1 and KATNAL1 disrupted numerous aspects of meiotic division and post-meiotic development, most of which are likely due to the compromised regulation of microtubule dynamics, including the formation of meiotic spindle, acrosome, nucleus, mitochondria sheath and flagella of sperm tail. These data add complementary information to previous studies of Katanin family during spermatogenesis and provide new foundations to understand the complex and coordinated cellular processes that spermatogenic cells undertake during their development.

Reviewer 1 Comments for the Author:

For most part of the manuscript, the phenotypes of mutant mice are clearly presented. The histological and electron microscopic images are of good quality. However, the mechanistic insights obtained from the data tend to be speculative. Some aspects of the manuscript are less clear and additional experiments may be needed for the conclusions drawn to be more convincing. These include:

1. It'll be helpful for the understanding of compensatory effects of Katanin subunits if their expression patterns during spermatogenesis could be introduced and compared, including that of KATNA1, KATNAL1, KATNAL2 and KATNB1. Since no obvious phenotype was observed for Katna1-CKO and dual deletion of Katna1 and Katnal1 showed more aggressive phenotypes than the deletion of Katnal1, what are the expression changes of the two genes in respective single mutants? If Katnal1-CKO showed severe phenotypes during meiosis and spermiogenesis, it may not suggest that “KATNA1 and KATNAL1 compensate for each other” (line 291) or “KATNA1 is dispensable for mammalian spermatogenesis” (line 117).

Response: As requested we have included additional analysis of *Katna1* and *Katnal1* mRNA in the

© 2023. Published by The Company of Biologists under the terms of the Creative Commons Attribution License (https://creativecommons.org/licenses/by/4.0/).

mouse testis of *Katnal1* and *Katna1* GCKO mice, respectively (New Fig. S6). No changes were observed in the expression of either subunit in the absence of the other, suggesting compensation is occurring at the level of severing activity. As the reviewer notes, more severe and additional abnormalities in meiosis and spermiogenesis, were observed in the *Katna1/al1*$^{GCKO/GCKO}$ mice compared to in the *Katnal1* single *GCKO* mice. Coupled with the absence of phenotype in the *Katna1* single GCKO mice, this provides direct functional evidence that KATNA1 and KATNAL1 can compensate for each other at a molecular level.

Consistent with this (and as detailed on lines 595-598 and now also on lines 77-79), we have previously shown that KATNB1-KATNA1 and KATNB1-KATNAL1 complexes associate with spindle microtubules during male meiosis (Dunleavy et al., 2021). Single cell analyses of the testis RNA transcriptome ((Ernst et al., 2019; Jung et al., 2019)) have also previously shown that both are expressed in meiosis and spermiogenesis. We have now included this information on line 595-598.

2. Were the axonemal structures examined for Katna1/Katnal1-DCKO mice using transmission electron microscopy?

Response: As requested, we have now included TEM of developing spermatid flagella in revised Fig 6I. This revealed axonemes formed in the Katna1/Katnal1 double GCKO model. The extremely low numbers of epididymal sperm in this model, however precluded a functional assessment or TEM analysis of mature sperm flagella.

3. Can the Co-IP/MS results be verified? For example, although the links to Actin cytoskeleton is interesting, would immunostaining of Actin reveal differences between wild type and KO sperm? Some Western blotting following Co-IP for proteins in the mentioned interactomes would be helpful.

Response: Validation of individual protein-protein interactions from the katanin testis interactome is beyond the scope of this already very large study. As such, and as per Reviewer 3’s suggestion, we have removed lines 440-486 from the results section wherein we had provided a detailed description of key individual proteins identified in the testis interactome IP. We have instead included a modified version of this text in the discussion and highlighted that future studies will be needed to validate and define these interactions.

4. The Stra8-Cre mouse lines used should be mentioned in the main text when introducing Katna1-GCKO and Katnal1-GCKO. Can Western blotting used to show the protein products in Katna1-GCKO mice if any (lines 106-107)?

Response: We have updated lines 112 and 133 of the main text to include reference to the Stra8-Cre mouse line. Unfortunately, the KATNA1 antibody is not suitable for western blotting i.e., it binds non-specifically to other proteins. It is does however appear specific when used for immunochemical methods as shown in Fig. S1E and for immunoprecipitation.

5.1 . Figure S3A, what the red arrow heads show? Indicate positions for sgRNAs, LoxPs, PCR primers for genotyping and scheme of resulting alleles; indicate deletion of ATPase and VPS4 domains? Off-target testing?

Response: we have updated the figure legend to clarify that the red arrows show the position of the *LoxP* insertion sites in the *Katnal1*$^{Flox/Flox}$ mice.

5.2 Figure S3E, how this staining is compared to that of Smith, et al., 2012 showing Sertoli specific expression? Is this due to the different antibodies used?

Response: different antibodies were used. The Smith et al., 2012 publication used an antibody generated by Covalab, whereas we generated a KATNAL1 antibody in house, which we have validated the specificity of with *Katnal1* knockout tissues.

6. The Materials and Methods section need to be more thorough and clearer, especially on, how the antibodies are used (Antibody use, Western blotting, Immunochemistry and Imaging sections should

© 2023. Published by The Company of Biologists under the terms of the Creative Commons Attribution License (https://creativecommons.org/licenses/by/4.0/).

be re- organized), how DSP is calculated, how the morphological defects in Katnal1-CKO are measured, how significant proteins are determined following MS. The statistics should be presented with s.d. and p values in the main text (lines 158-167, lines 315-316).

Response: As requested, we have reorganised and updated the methods and main text to clarify the requested details.

Some minor concerns:

1. Instead of using "GCKO" in most of the figures, specific genes affected should be shown, for example, Katnal1-GCKO, in order to improve the readability.

Response: we have updated figures accordingly.

2. Lines 146-148 described Sertoli-only tubules, which is hardly shown in Fig.1F.

Response: we have removed this sentence.

3. Indicate midbodies in Fig. 2Ac, g and Fig. 5Ag, k (lines 348-349) with arrows;

Response: we have updated figures accordingly.

4. Line 1150, blue or green arrowheads?

Response: it should have stated green arrowheads. We have corrected the figure legend accordingly.

5. Line 420, to show the significance determined by what criteria?

Response: we have updated the text to clarify that significance was determined using a two-sided t-test, with P values below <0.05 defined as significant.

***** Reviewer 2 Advance Summary and Potential Significance to Field:

The paper by Jessica Dunleavy et al. entitled "Katanin A-subunits KATNA1 and KATNAL1 act cooperatively in mammalian meiosis and spermiogenesis to achieve male fertility" describes the establishment and analysis of Katna1 and Kantal1 deficient mouse lines. The authors report many pathophysiological changes and perform a wide set matings in order to decipher common and compensatory effects of microtubule severing proteins.

Reviewer 2 Comments for the Author:

While Dunleavy et al. report interesting phenotypes, which adds to the understanding of the genes required for spermatogenesis the authors need to address some points before the ms should be considered for publication. Further, the reviewer would like to mention, that reporting on aberrations of sperm in epididymis from such animals as primary effect of the mutation is tricky. Obviously, spermatogenesis and spermiogenesis are affected at earlier stages leading to initiation of cell death – hence, what the authors are observing might represent a secondary effect. This option should be mentioned and discussed.

Response: If I understand, we are in agreement. As detailed more thoroughly below, our data strongly suggests that the abnormalities in the epididymal sperm arise due abnormal assembly of key structures during spermiogenesis. Nevertheless, the mice are infertile and a description of abnormalities in epididymal sperm is clinically relevant phenotypic information regardless of the primary origin.

In detail:

1. line 133 – Fig 1D does not show apoptotic germ cells as stated in the text. Pls. revise.

© 2023. Published by The Company of Biologists under the terms of the Creative Commons Attribution License (https://creativecommons.org/licenses/by/4.0/).

Response: this should have referred to Fig 2D, this data has now been moved to Fig S3F and the text (now line 143) revised.

2. line 158 ff. paragraph describing morphologically abnormal sperm in Katnl1 gko mice. To the reviewer's opinion, this defect could very well be a secondary effect, since many murine models showing problems in spermiogenesis show malformed sperm owing to sperm degradation in the epididymis. Either, the authors water the claim down and include the possibility of this being a secondary effect, or they present pictures of malformed sperm in the testes.

Response: We appreciate this opinion, as detailed in Fig 3E and F, we show that the origins of the sperm malformations, including defects in the sperm head-to-tail coupling apparatus and defects in sperm head shape arise during spermiogenesis i.e., the defect originates in the testis and remains present in epididymal sperm

We have also included additional EM data to inform the development of the flagella in spermatids in revised Fig 3D. This reveals that defects in the flagella become more overt during transit through the epididymis, possibly as a consequence of the application of sheering forces during the transit process. These data suggest that the integrity of the sperm tail is compromised in the *Katnal1* GCKO mice. We have updated lines 268-282 of the results and 638-639 of the discussion to reflect this interpretation.

3.) Figures are sometimes not listed in proper succession, e.g. Line 142 -Fig 1G and line 146 Fig E,F – There are more of such examples – this makes reading difficult. Pls. re-organize the figures accordingly.

Response: we have updated the text and reorganised figures accordingly.

4.) line 164 – "the ability for motility" sounds odd – why not "reduction in motility"
Response: we have updated the text accordingly.

5.) line 169 – the claim is too general, it needs to be more specific.

Response: we have updated the lines 184-185 to state: 'Collectively these phenotypes reveal germ cell derived KATNAL1 is required for normal sperm number, morphology and motility and optimal male fertility'

6.) Fig 2A – would benefit from a fluorescent staining method, alternatively, please add some (bigger) arrowheads to pinpoint what you describe in order for the non-meiosis aficionado get your point.

Response: we have added additional arrowheads and labels to Fig 2, as well as Fig 5, to assist in interpretation by readers.

7.) Fig. 2D seems not mentioned in the text.

Response: as above, this data has now been moved to Fig S3F and the text corrected on line 143 to refer to this data.

8.) Fig. 2 E-J are only mentioned in one half sentence together with Fig 2A (line 184). Either explain to the reader what is there to see or move them to supplemental.

Response: these have been moved to Fig S3.

9.) Line 193 and 198 the reviewer reads some relative terms such as "often observed" or "a subset of the resulting spermatids". Could the authors try to count these observations and add them to the (then amended) text?

Response: we have quantified these defects in chromosome misalignment and occurrence of binucleated spermatids and added them in to Fig 2.

© 2023. Published by The Company of Biologists under the terms of the Creative Commons Attribution License (https://creativecommons.org/licenses/by/4.0/).

10.) Fig. 3A and B – is a blurry and too little enlarged photograph. The reviewer suggests to take pictures at higher magnification and probably add the overview picture as supplemental material.

Response: we have updated Fig 3A with images taken at a higher magnification.

11.) Line 226 ff. Fig. 3C. Again, the reviewer stresses the point that this could be a secondary effect of the degradation process. This possibility must be mentioned, when presenting the data. Alternatively, sections from testes demonstrating such defects would suffice to make the point.

Response: please see our response to comment 2.

12.) Line 235. The authors use here the term "wild-type" – to be correct it is material from flox/flox – mice. They might be considered "controls" but the term wild-type might be misleading.

Response: we have updated the text accordingly.

13.) Line 246 ff. refers to point 11 – consider revising owing to this potentially being a secondary defect.

Response: please see our response to comment 2.

14.) Line 261 – The authors state "In some cells.." – please quantify "some" to substantiate the claim and give the reader an impression of the extent of the sperm heads altered.

Response: to more accurately quantitate the claim that normal HTCAs were rare in Katnal1 GCKO mice we used the HTCA marker SUN5 in Fig S4D,E. We have now included additional data on lines 302-303 quantifying our assessment of HTCA morphology via TEM – noting that this is an extremely laborious process. Of spermatids that had visible HTCA structures (basal plate, capitulum, segmented columns, basal body) via TEM, 64% were abnormal in Katnal1 GCKO compared to 0% in Katnal1 Flox.

15.) Line 265 – SUN5 Immunofluorescence must be repeated in order to give a result, which one can interpret. At present the picture is too dark/staining too weak to be able to interpret.

Response: this is likely a display issue during the pdf production process. To circumvent this problem we have replaced the representative images with ones taken at higher magnification. We have also uniformly adjusted the brightness and contrast of the representative images and added arrows to allow easier interpretation.

16.) Line 270-271 – this claim seems not backed by the data – could be presented as speculation.

Response: we have removed this sentence.

17.) Line 282 "dissembled" – should it read "disassembled"?

Response: we have updated the text accordingly.

18.) Line 287 Fig 3F – could the pictures of control and the mutant be taken with the same intensity/white balance in order to be able to compare the staining. Also, manchette development is really much better presented by using fluorescent labelling (PNA-FITC or comparable). The reviewer thinks that replacing the present pictures by fluorescent ones would improve the ability to judge the claims made by the authors.

Response: The images were taken with the same white balance, the amount and localisation of alpha tubulin however differs between genotypes. We have however replaced some panels to improve ease of interpretation.

We disagree that a further method is needed to assess manchette development. This phenotype has been clearly demonstrated using both electron microscopy and light microscopy techniques.

© 2023. Published by The Company of Biologists under the terms of the Creative Commons Attribution License (https://creativecommons.org/licenses/by/4.0/).

Moreover, DAB- staining was chosen to allow precise stage-dependent assessment of manchette migration as it permits the visualisation of nuclear and other cell structures. Given that there is a dysregulation of spermiogenesis in this model, staging relies on assessment of cell types such as spermatogonia and spermatocytes, which is most reliably achieved via light microscopy.

We note PNA-FITC stains the acrosome not the manchette.

19.) Line 291 ff – paragraph discusses the compensation of both proteins deleted. However, in technical terms, this claim is made by assessing anatomy and morphology. There are no molecular data such as qRT- PCR or Western Blot in order to look for levels of transcripts or proteins in the respective ko mice. Maybe there is a compensation i.e. upregulation, but this is not sufficient. The authors should consider mention this in the text or provide experiments to substantiate the claim.

Response: as detailed in our response above to Reviewer 1, our data provide direct functional evidence of compensation between KATNA1 and KATNAL1 in the mouse testis. We have now included the requested molecular data in Fig S6. Please see response to comment 1 of Reviewer 1 for details.

We note that our claims are also confirmed by functional data at both a cellular and physiological level e.g. sperm motility and fertility respectively.

20.) line 304 – the authors should make it clear that with this sentence “No aspect..” they refer not only to their results presented here, but also to the previous publication they mention before.

Response: we have updated the text to include a reference to the previous publication.

21.) line 310 – “Successful” ablation of…Please delete “successful” since it is meaningless.

Response: we have updated the text accordingly.

22.) line 321 – Fig. 4g-H is too small magnification to really see all the details. A higher magnification must be provided. Further, the arrowheads are too small and the colors of the arrows is unfortunate so that they are only hard to see. Please revise this part of the figure.

Response: we have updated the figure and arrowheads accordingly.

23.) line 355-357 – the authors claim that the frequency of binucleated spermatids…. was similar to….The reviewer wonders where to find the data (numbers) and the respective statistics for this claim. Please add.

Response: thank you for this suggestion, we have quantified these defects, and added them in to Fig 2 and Fig 5. Quantification revealed that the frequency of binucleated spermatids were three-fold higher in the *Katna1/al1* GCKO mice as compared to the Katnal1 GCKO mice. We updated our interpretation to reflect this.

24.) line 359: typo spermiogenesis revealed

Response: we have updated the text accordingly.

25.) line 360 – the analysis of acrosome biogenesis (Fig 6 A,B) would benefit from a immunofluorescent staining. Alternatively, higher mag insets could make the point as well. Blue arrowhead is hard to see – consider other color.

Response: As per the reviewer’s suggestion we have included an additional higher magnification panel of the PAS-stained testis sections in Fig 6 (new panel B) to further demonstrate the acrosome phenotypes. We have also changed the arrowhead colour in Fig 6B (now Fig 6C).

As for the manchette analysis above, our preference is to use PAS staining to analyse acrosome structure at a light microscopic level where possible as it allows an assessment of acrosome structure in the context of other organelles and surrounding cell types. The light microscopic

© 2023. Published by The Company of Biologists under the terms of the Creative Commons Attribution License (https://creativecommons.org/licenses/by/4.0/).

observations were confirmed using electron microscopy.

26.) line 389 Fig 6G manchette analysis would benefit from immunofluorescent labelling

Response: please see response to comment 19 above.

27.) line 414 ff – the authors finally perform an IP and a MS analysis to detect potential interaction partners. To the reviewer this paragraph, while interesting has some shortcomings. First, none of the detected proteins are validated by a further experiment (CoIP, reverse IP), second the candidate mentioned before SUN5 (see point 7) seems not detected or is at least not mentioned in the main text. Second – the MS analysis detects protein (fragments) which are pulled down in such an experiment. However, without further biochemical analyses, one cannot state that protein x "is bound to" KATNA1 etc. Since there is the potential, that the pull- down reflects a larger protein complex "bound" can not be claimed here. Further, although, the authors filtered the data, there is always the potential of a backgound noise, which one only gets rid off by performing further experiments to validate the interaction. So, in sum, the last part appears rather weak and should be strengthened by validation experiments. Alternatively, since it might extend beyond the scope of this manuscript, the MS data could be considered to be left out to serve as a starting point for a (more biochemical) follow up study.

Response: We accept this comment. As per our response to Reviewer 1 validation of individual protein-protein interactions from the katanin testis interactome is beyond the scope of this already very large study. As such, and as per Reviewer 3's suggestion, we have removed lines 440-486 from the results section wherein we had provided a detailed description of key individual proteins identified in the testis interactome IP. We have instead included a modified version of this text in the discussion and highlighted that future studies will need to validate and defined these interactions.

We note that while SUN5 was found to be mislocalised in Katnal1 GCKO mice, it was not identified as a putative katanin binding partner in our IP data. SUN5 was used a marker of the sperm HTCA to confirm our observation by EM that HTCA's were abnormal Katnal1 GCKO mice. Mislocalisation of SUN5 is likely a consequence of the abnormal HTCA development as opposed to a due to a direct interaction with KATNAL1.

******* Reviewer 3 Advance Summary and Potential Significance to Field:**

The authors have conducted a study on the function of Katanin A-subunit protein inmale germ cells using conditional KO mice. The data presented in the study have been diligently analyzed and the results are quite convincing. However, the manuscript could benefit from some trimming as it is quite lengthy and some parts tend to blur the main focus. If the authors remove unnecessary parts of the sentences, the paper would become more readable and effective.

We have edited the manuscript accordingly, while still retaining enough information for non-experts to be able to understand the findings.

Reviewer 3 Comments for the Author:

Major points

1. On line 221, the authors mention that Katnal1 KO has abnormal sperm tails, but no quantitative data is provided. It would be helpful to have a clear illustration of the abnormal ratio of sperm tail in Katnal1 KO in Fig. 1J. Additionally, the KO's sperm midpiece defects seem to be around 10%, including coiled midpiece and mitochondrial sheath defects. This ratio is not significantly high. While the authors noted sperm tail abnormalities in Fig. 3C, these data were obtained from the cauda epididymis. Therefore, to conclude that KATNAL1 is essential for "axoneme doublet formation" and "ODF/Mitochondrial sheath formation," the author must observe those organelles during spermiogenesis (testis). Since sperm obtained some stress during epididymal migration, and sperm in Fig. 3C may reflect secondary effects. To disprove this point, the author must place TEM pictures for step 16 spermatids in Fig. 3C. My guess is that abnormalities in step 16 spermatid tail are scarce. In that case, it would be best not to make many mentions of the sperm tail since it is far

© 2023. Published by The Company of Biologists under the terms of the Creative Commons Attribution License (https://creativecommons.org/licenses/by/4.0/).

from the main story of the paper.

Response: the quantitative data had already been mentioned earlier including functional abnormalities (sperm motility data on lines 179-184), and the morphological defects described in epididymal sperm on lines 172-179 (abnormal sperm head reticulation, decapitation, sperm tail mid-piece coiling and mitochondrial sheath defects). As the reviewer notes only a subset of sperm tails have morphological abnormalities that are discernible by light microscopy, however as shown by our analysis this is a statically significant increase compared to controls. We also note the functional defects are substantial with a 52.9% reduction in progressively motile sperm and sterility at a physiological level.

As requested, we have also included additional EM data to assess the development of the flagella in spermatids in Fig 3D. This reveals that defects in the flagella become most apparent after transit through the epididymis, suggesting that the integrity of the sperm tail is compromised in the *Katnal1* GCKO mice. We have updated lines 268-282 of the results and 638-639 of the discussion to reflect this interpretation.

2. Calculate the proportion of "misaligned/lagging chromosomes" in Fig. 5B, "binucleated round spermatids" in Fig. 5C, and abnormal acrosome vesicles in Fig. 6A? These findings are critical to this research paper.

Response: As requested we have quantified these defects, and added them in to Fig 5 and Fig 6.

3. Regarding sentences 440-486, they mostly constitute the discussion section. If you intend to incorporate them into the results section, it would be necessary to conduct further experiments. Thus, I suggest that these sentences be excluded from the paper.

Response: as per the reviewer's suggestion we have removed this text from the results and incorporated a modified version of this text in the discussion instead.

Minor points

In the Results section, there is no reference to the use of Stra8-Cre Tg mice. These mice were utilized in the creation of all conditional KO mice mentioned in the study. It would be helpful to include this information in the Results section to aid the reader's comprehension.

Response: We have updated lines 112 and 133 of the main text to include reference to the Stra8-Cre mouse line.

The genes Katna1 and Katnal1 have such similar names that it can be difficult to tell them apart. While I understand that these are official gene symbols, could simpler names be used in the paper? The current names have caused confusion for me and other readers, adding unnecessary stress.

Response: while we appreciate that they have similar names, we have used the official gene/protein nomenclature as per the journal guidelines.

In line 138, based on the gathered data, would it be more appropriate to use "spermatogenesis" instead of "spermiogenesis" in the sentence "This reveals that gem cell derived KATNAL1 is required for optimal spermiogenesis and~"?

Response: we have updated the text accordingly.

Line 155-156, "These data reveal that KATNAL1 plays an essential role in MT regulation in both Sertoli and germ cells" I think KATNAL1 is not essential for Sertoli cells from your data. spermiogenesis and~"?

Response: we have updated the text (now lines 169-170) to clarify that we are referring to the combination of our data and the previous data characterising the role of KATNAL1 in Sertoli cells.

Line 205-209, I think this paragraph can be removed.

© 2023. Published by The Company of Biologists under the terms of the Creative Commons Attribution License (https://creativecommons.org/licenses/by/4.0/).

Response: we have updated the text accordingly.

Line 303-306, Please include actual data from mating tests of Katna1/al2 double KO mouse line.

Response: we have previously shown that single *Katnal2* deletion results in male sterility, as such we did not do mating tests for the *Katna1/al2* double deletion mice.

Line 351, Is Fig. 5Ag,k is correct? I think it should be Fig.5h,i.

Response: we have corrected the text, the correct panel was Fig 5Ca,c.

Line 351-353, I think this sentence can be combined with the previous sentence.

Response: we appreciate the suggestion, however as they describe two separate processes, we think two sentences provides better clarity.

Line 491, 492, “for the first time”, “first”. I think these expressions are not suitable for a research paper.

Response: we have updated the text accordingly.

Fig. 1C, 4E, S2C, S5E, DSP is only spelled out in Materials and Methods. Please spell out in Line 134.

Response: we have updated text on line 118 to define this abbreviation.

Fig. 3Cc, Fig. 3Ca-c should be pictures for midpiece, but Fig. 3Cc is a picture for principal piece.

Response: Fig 3Cc is included to show the abnormalities in ODF and axoneme doublet number and localisation. We have updated the figure legend to clarify the identity of each tail cross-section.

Fig. 3F, In the process of spermiogenesis, does the Manchette of Katnal1 KO disappear? If it disappears, please put pictures until it disappears.

Response: we have updated the figure accordingly.

Fig.4, There are no pictures for sperm morphology of Katna1/al1 KO. This data is thought to be important.

Response: we have now included this data in Fig 4Q

References

Dunleavy, J. E. M., O'Connor, A. E., Okuda, H., Merriner, D. J. and O'Bryan, M. K. (2021). KATNB1 is a master regulator of multiple katanin enzymes in male meiosis and haploid germ cell development. *Development* **148**, dev199922.

Ernst, C., Eling, N., Martinez-Jimenez, C. P., Marioni, J. C. and Odom, D. T. (2019). Staged developmental mapping and X chromosome transcriptional dynamics during mouse spermatogenesis. *Nature communications* **10**, 1251.

Jung, M., Wells, D., Rusch, J., Ahmad, S., Marchini, J., Myers, S. R. and Conrad, D. F. (2019). Unified single-cell analysis of testis gene regulation and pathology in five mouse strains. *Elife* **8**, e43966.

Second decision letter

MS ID#: DEVELOP/2023/201956

© 2023. Published by The Company of Biologists under the terms of the Creative Commons Attribution License (https://creativecommons.org/licenses/by/4.0/).

MS TITLE: Katanin A-subunits KATNA1 and KATNAL1 act co-operatively in mammalian meiosis and spermiogenesis to achieve male fertility

AUTHORS: Jessica EM Dunleavy, Maddison Graffeo, Kathryn Wozniak, Anne O'Connor, D. Jo Merriner, Joseph Nguyen, Ralf B Schittenhelm, Brendan Houston, and Moira K O'Bryan

I have now received all the referees reports on the above manuscript, and have reached a decision. The referees' comments are appended below, or you can access them online: please go to BenchPress and click on the 'Manuscripts with Decisions' queue in the Author Area.

The overall evaluation is positive and we would like to publish a revised manuscript in Development, provided that the referees' comments can be satisfactorily addressed. Please attend to all of the reviewers' comments in your revised manuscript and detail them in your point-by-point response. If you do not agree with any of their criticisms or suggestions explain clearly why this is so. If it would be helpful, you are welcome to contact us to discuss your revision in greater detail. Please send us a point-by-point response indicating your plans for addressing the referees' comments, and we will look over this and provide further guidance.

Reviewer 1

Advance summary and potential significance to field

The authors elucidated the functional roles of a family of important microtubule severing enzyme, Katanin using mouse genetics, as well as cell biological and biochemical experiments. Their data showed that the catalytic subunits KATNA1 and KATNAL1 of Katanin complex (composed of catalytic A and regulatory B subunits) play essential roles during meiosis and spermiogenesis in mice. Specifically, deletion of KATNAL1 or both KATNA1 and KATNAL1 disrupted numerous aspects of meiotic and post-meiotic development, most of which are likely due to the compromised regulation of microtubule dynamics, including the formation of meiotic spindle and chromosome segregation of spermatocytes, acrosome formation, nucleus, mitochondria sheath and flagella formation of haploid spermatids, leading to eventual male sub- or infertility. These data add complementary information to previous studies of Katanin family during spermatogenesis and provide new foundations to understand the complex and coordinated cellular processes that spermatogenic cells undertake during their development.

Comments for the author

In this revision, the authors have included new data, re-organized some of the contents and improved the clarity of the manuscript.
Some minor points that can be addressed in the main text:
1) One concern is that the presented data show that mutations of either KATNA1 or KATNAL1 gene mutation elicited different phenotypes, and double knockout mice had more severe defects, which suggest a possibility that the two proteins play synergistic roles during meiosis and spermiogenesis, instead of a compensatory function (e.g., lines 372-373, lines 484-485). This should be taken into consideration when discuss the findings.
2) the sub-cellular localizations of KATNA1 and KATNAL1 proteins, do they associate with MT or spindle poles?
3) Fig 3H-I, what are the electron dense materials surrounding the nucleus?
4) Re-phrase line 410;
5) line 458, Fig S7E-G?
6) Proteins identified in the IP/MS experiments not necessarily mean they are proteins interacting with each other (as described in the Results and Discussion).

Reviewer 2

Advance summary and potential significance to field

© 2023. Published by The Company of Biologists under the terms of the Creative Commons Attribution License (https://creativecommons.org/licenses/by/4.0/).

Think I already wrote some sentences previously

Comments for the author

All my comments and criticism has been addressed. I do have no further points which need to be addressed.

Reviewer 3

Advance summary and potential significance to field

The authors addressed my concerns. The revised manuscript should be published.

Comments for the author

I have no further comments except for the below typo.

L189: double space

L220, L237: Abnormalities

L238: epididymal

L240, 241, 402, 1196: asterisks

L245: presence

L252: There is an unnecessary space.

L343, 722: chromosomes

L376: analysis

L483: manner is duplicated.

L665-666: mice is duplicated.

L690: no space before parentheses

L700, 741: Briefly

L700: quantification

L712: assessment

L714: abnormal

L715: sheath

L757: Fluorescent

L767: antibodies

L786: a Alexa > an Alexa

L1133: indicates

L1134: piece

© 2023. Published by The Company of Biologists under the terms of the Creative Commons Attribution License (https://creativecommons.org/licenses/by/4.0/).

L1172: indicate

Second revision

Author response to reviewers' comments

Response to reviewers
We thank the reviewers for the time and effort they have put into reviewing our manuscript. We have addressed each comment below and trust that the manuscript is now acceptable for publication in Development.

Reviewer 1 Advance Summary and Potential Significance to Field:

The authors elucidated the functional roles of a family of important microtubule severing enzyme, Katanin, using mouse genetics, as well as cell biological and biochemical experiments. Their data showed that the catalytic subunits KATNA1 and KATNAL1 of Katanin complex (composed of catalytic A and regulatory B subunits) play essential roles during meiosis and spermiogenesis in mice. Specifically, deletion of KATNAL1 or both KATNA1 and KATNAL1 disrupted numerous aspects of meiotic and post-meiotic development, most of which are likely due to the compromised regulation of microtubule dynamics, including the formation of meiotic spindle and chromosome segregation of spermatocytes, acrosome formation, nucleus, mitochondria sheath and flagella formation of haploid spermatids, leading to eventual male sub- or infertility. These data add complementary information to previous studies of Katanin family during spermatogenesis and provide new foundations to understand the complex and coordinated cellular processes that spermatogenic cells undertake during their development.

Reviewer 1 Comments for the Author:

In this revision, the authors have included new data, re-organized some of the contents and improved the clarity of the manuscript.

Some minor points that can be addressed in the main text:

1) One concern is that the presented data show that mutations of either KATNA1 or KATNAL1 gene mutation elicited different phenotypes, and double knockout mice had more severe defects, which suggest a possibility that the two proteins play synergistic roles during meiosis and spermiogenesis, instead of a compensatory function (e.g., lines 372-373, lines 484-485). This should be taken into consideration when discuss the findings.

Response: the KATNA1 single knockout mouse model did not have any abnormal phenotypes. We believe that this discounts the possibility that the two proteins play purely synergistic roles as opposed to compensatory roles. As if the more severe phenotypes in the double GCKO were due to a synergistic role, we would have expected to see abnormal phenotypes in the KATNA1 single knockout mouse, as well as in KATNAL1 single knockout mouse.

This confusion may have arisen due to the previous wording of lines 303-305 – which stated: ‘By contrast double germ cell knockout of *Katna1* and *Katnal1* resulted in a significantly worse male fertility phenotype than seen in either individual knockout, especially in meiosis’. We have updated this line to avoid any confusion.

2) the sub-cellular localizations of KATNA1 and KATNAL1 proteins, do they associate with MT or spindle poles?

Response: They associate with spindle MT fibres. This information is detailed on line 495.

3) Fig 3H-I, what are the electron dense materials surrounding the nucleus?

© 2023. Published by The Company of Biologists under the terms of the Creative Commons Attribution License (https://creativecommons.org/licenses/by/4.0/).

Response: We believe what the reviewer is referring to is the nuclear lamina, which an intermediate filament-based structure which lines the inner surface of the nuclear membrane. This appears similar between genotypes and is not the focus of these panels, which are to look at the position of perinuclear ring of the manchette.

4) Re-phrase line 410;

Response: We have rephrased this for clarity (now lines 406-409)

5) line 458, Fig S7E-G?

Response: Thank you for noticing this error, it has been corrected to Fig S7E-J.

6) Proteins identified in the IP/MS experiments not necessarily mean they are proteins interacting with each other (as described in the Results and Discussion).

Response: We have further modified the language reflect this. Moreover, on lines 602-603

******* Reviewer 2 Advance Summary and Potential Significance to Field:**
Think I already wrote some sentences previously

Reviewer 2 Comments for the Author:
All my comments and criticism has been addressed. I do have no further points which need to be addressed.

******* Reviewer 3 Advance Summary and Potential Significance to Field:**
The authors addressed my concerns. The revised manuscript should be published.

Reviewer 3 Comments for the Author:

I have no further comments except for the below typo.

Response: We thank reviewer 3 for noticing the below typos, they have now been corrected.

L189: double space

L220, L237: Abnormalities

L238: epididymal

L240, 241, 402, 1196: asterisks

L245: presence

L252: There is an unnecessary space.

L343, 722: chromosomes

L376: analysis

L483: manner is duplicated.

L665-666: mice is duplicated.

L690: no space before parentheses

L700, 741: Briefly

© 2023. Published by The Company of Biologists under the terms of the Creative Commons Attribution License (https://creativecommons.org/licenses/by/4.0/).

L700: quantification

L712: assessment

L714: abnormal

L715: sheath

L757: Fluorescent

L767: antibodies

L786: a Alexa > an Alexa

L1133: indicates

L1134: piece

L1172: indicate

Third decision letter

MS ID#: DEVELOP/2023/201956

MS TITLE: Katanin A-subunits KATNA1 and KATNAL1 act co-operatively in mammalian meiosis and spermiogenesis to achieve male fertility

AUTHORS: Jessica EM Dunleavy, Maddison Graffeo, Kathryn Wozniak, Anne O'Connor, D. Jo Merriner, Joseph Nguyen, Ralf B Schittenhelm, Brendan Houston, and Moira K O'Bryan
ARTICLE TYPE: Research Article

I am happy to tell you that your manuscript has been accepted for publication in Development, pending our standard ethics checks.

© 2023. Published by The Company of Biologists under the terms of the Creative Commons Attribution License (https://creativecommons.org/licenses/by/4.0/).
